# Supplementary material for: Conformation-dependent degradation of thermally activated delayed fluorescence materials bearing cycloamino donors
Source: Commun Chem. 2020 May 1;3:53. doi: 10.1038/s42004-020-0303-4 (PMC9814945; doi:10.1038/s42004-020-0303-4)
Supplement: Supplementary file 2 — Supplementary Information [file 42004_2020_303_MOESM2_ESM.pdf]

**Supplementary Information for**

# Conformation-Dependent Degradation of Thermally Activated Delayed Fluorescence Materials Bearing Cycloamino Donors

*Sanju Hwang et al.*

**Supplementary Table 1. Photophysical data for the donor–TRZ vompounds**

| | $E_{S1}$ (eV)[a] | $E_{T1}$ (eV)[b] | $\Delta E_{1ICT-3ICT}$ (eV)[c] | $\Delta E_{3LE-T1}$ (eV)[d] | $\Phi_{PF}$[e] | $\Phi_{DF}$[f] | $\Phi_{ISC}$[g] | $\Phi_{rISC}$[h] |
|---|---|---|---|---|---|---|---|---|
| Cbz-TRZ | 2.98 | 2.69 | 0.29 | 0.42 | 1.00 | 0 | 0 | 0 |
| AZP$^{ax}$-TRZ | 3.18 | 2.66 | 0.15 | 0.51 | 0.20 | 0 | 0.80 | 0 |
| AZP$^{eq}$-TRZ | 2.65 | 2.61 | 0.04 | 0.41 | | | | |
| DMAC$^{ax}$-TRZ | 3.15 | 2.63 | 0.13 | 0.53 | 0.52 | 0.18 | 0.48 | 0.38 |
| DMAC$^{eq}$-TRZ | 2.51 | 2.50 | 0.01 | 0.52 | | | | |
| PXZ$^{ax}$-TRZ | 3.21 | 2.69 | 0.13 | 0.47 | 0.29 | 0.41 | 0.71 | 0.58 |
| PXZ$^{eq}$-TRZ | 2.13 | 2.12 | 0.01 | 0.90 | | | | |
| PTZ$^{ax}$-TRZ | 3.24 | 3.10 | 0.14 | 0.45 | 0.11 | 0.29 | 0.89 | 0.33 |
| PTZ$^{eq}$-TRZ | 2.41 | 2.40 | 0.01 | 0.62 | | | | |

[a]Calculated energy of the $S_1$ state (TD–CAM–B3LYP/6–311+G(d,p)//CAM–B3LYP/6–311+G(d,p)). [b]Calculated energy of the $T_1$ state (TD–CAM–B3LYP/6–311+G(d,p)//CAM–B3LYP/6–311+G(d,p)). [c]Calculated energy difference between the singlet ($^1$ICT) and triplet ($^3$ICT) transition states. [d]Calculated energy difference between the $^3$LE state and the $T_1$ state. [e]Quantum yield for prompt fluorescence. [f]Quantum yield for delayed fluorescence. [g]Quantum yield for ISC. $\Phi_{ISC} = 1 - \Phi_{PF}$. [h]Quantum yield for rISC. $\Phi_{rISC} = \Phi_{DF}/\Phi_{ISC}$.

**Supplementary Table 2. Calculated geometric and electronic parameters of the pseudo-axial and pseudo-equatorial forms of the donor–TRZ compounds[a]**

| | C–N bond length; $S_0/S_1$ (Å)[b] | $BDE_{C–N}$ (eV)[c] | $BDE_{C–C}$ (eV)[d] | $BDE_{C–N} - E_{1ICT}$ (eV)[e] | $BDE_{C–C} - E_{1ICT}$ (eV)[f] |
|---|---|---|---|---|---|
| Cbz-TRZ | 1.418/1.451 | 3.50 | 4.81 | 0.52 | 1.83 |
| AZP$^{ax}$-TRZ | 1.396/1.380 | 3.12 | 4.93 | −0.06 | 1.75 |
| AZP$^{eq}$-TRZ | 1.442/1.472 | 2.81 | 4.80 | 0.16 | 2.15 |
| DMAC$^{ax}$-TRZ | 1.406/1.396 | 2.87 | 4.92 | −0.28 | 1.77 |
| DMAC$^{eq}$-TRZ | 1.437/1.463 | 2.95 | 4.79 | 0.44 | 2.28 |
| PXZ$^{ax}$-TRZ | 1.416/1.423 | 2.57 | 4.91 | −0.64 | 1.70 |
| PXZ$^{eq}$-TRZ | 1.433/1.460 | 2.69 | 4.79 | 0.56 | 2.66 |
| PTZ$^{ax}$-TRZ | 1.410/1.401 | 2.74 | 4.92 | −0.50 | 1.68 |
| PTZ$^{eq}$-TRZ | 1.438/1.465 | 2.72 | 4.79 | 0.31 | 2.38 |

[a]CAM-B3LYP/6–311+G(d,p). [b]Lengths of the C–N bond between the donor and TRZ of the donor–TRZ compounds in their ground ($S_0$) and first singlet excited ($S_1$) states. [c]The dissociation energy of the C–N bond between the donor and TRZ of the compounds in their ground states. [d]The dissociation energy of the C–C bond between the phenyl and triazine of the compounds in their ground states. [e]Difference between the $BDE_{C–N}$ and the singlet intramolecular charge-transfer ($^1$ICT) transition state energy. [f]Difference between the $BDE_{C–N}$ and the $^1$ICT transition state energy.

**Supplementary Table 3. Summary of electroluminescence performances of the devices employing the donor–TRZ compounds as dopants**[a]

| | $V_{200\ nit}$ (V)[b] | $J_{200\ nit}$ (mA cm$^{-2}$)[c] | EQE (%)[d] | | PE (lm W$^{-1}$)[e] | | LE (cd A$^{-1}$)[f] | |
|---|---|---|---|---|---|---|---|---|
| | | | 200 cd m$^{-2}$ | max | 200 cd m$^{-2}$ | max | 200 cd m$^{-2}$ | max |
| AZP-TRZ | 8.8 | 1.07 | 1.5 | 1.5 | 0.68 | 0.84 | 1.89 | 1.99 |
| DMAC-TRZ | 7.3 | 0.93 | 8.2 | 8.5 | 9.18 | 9.22 | 21.25 | 21.94 |
| PXZ-TRZ | 6.6 | 0.43 | 14.1 | 14.1 | 21.91 | 22.28 | 46.13 | 46.17 |
| PTZ-TRZ | 7.1 | 0.51 | 12.4 | 13.0 | 17.37 | 20.10 | 39.47 | 41.59 |

[a]ITO / DNTPD (60 nm) / BPBPA (20 nm) / PCzAC (10 nm) / mCBP:10 wt % dopant (30 nm) / DBF-Trz (5 nm) / ZADN (30 nm) / LiF (1 nm) / Al. [b]Voltage at 200 cd m$^{-2}$. [c]Current density at 200 cd m$^{-2}$. [d]External quantum efficiency. [e]Power efficiency. [f]Luminous efficiency.

**Supplementary Table 4. Crystallographic data for AZP-TRZ**

| | **AZP-TRZ** |
|---|---|
| Empirical formula | $C_{35}H_{26}N_4$ |
| Formula weight | 502.60 |
| Temperature (K) | 223(2) |
| Wavelength (Å) | 0.71073 |
| Crystal system/space group | Monoclinic, $P2_1/c$ |
| Unit cell dimensions | |
| *a* (Å) | 19.8371(6) |
| *b* (Å) | 9.7227(3) |
| *c* (Å) | 26.6431(9) |
| $\alpha$ (°) | 90.00 |
| $\beta$ (°) | 91.6354(12) |
| $\gamma$ (°) | 90.00 |
| Volume (Å$^3$) | 5136.6(3) |
| Z | 8 |
| Calculated density (g/cm$^{-3}$) | 1.300 |
| Absorption coefficient (mm$^{-1}$) | 0.077 |
| Reflections collected | 196332 |
| Independent reflections [*R*(int)] | 10166 [0.1569] |
| Refinement method | Full-matrix |
| | least-squares on $F^2$ |
| Data/restraints/parameters | 10166/0/703 |
| Goodness-of-fit on $F^2$ | 1.044 |
| Final *R* indices | $R_1$ = 0.0481, |
| [$I > 2$sigma($I$)] | $wR_2$ = 0.0889 |
| *R* indices (all data) | $R_1$ = 0.0958, |
| | $wR_2$ = 0.1046 |
| Largest difference peak and hole (e/Å$^3$) | 0.230 and −0.182 |

**Supplementary Table 5. Selected bond distances (Å) for AZP-TRZ**

| bond distances (Å) | | | | | | | |
|---|---|---|---|---|---|---|---|
| C(1)-N(1) | 1.334(2) | C(16)-C(17) | 1.389(2) | C(34)-C(35) | 1.382(2) | C(51)-C(56) | 1.384(2) |
| C(1)-N(3) | 1.338(2) | C(17)-C(18) | 1.379(2) | C(36)-N(7) | 1.334(2) | C(51)-C(52) | 1.385(2) |
| C(1)-C(4) | 1.482(2) | C(18)-C(19) | 1.398(2) | C(36)-N(5) | 1.338(2) | C(52)-C(53) | 1.376(3) |
| N(1)-C(2) | 1.338(2) | C(19)-N(4) | 1.393(2) | C(36)-C(39) | 1.482(2) | C(53)-C(54) | 1.392(2) |
| C(2)-N(2) | 1.340(2) | C(19)-C(20) | 1.400(2) | N(5)-C(37) | 1.337(2) | C(54)-N(8) | 1.393(2) |
| C(2)-C(10) | 1.479(2) | C(20)-C(21) | 1.370(2) | C(37)-N(6) | 1.338(2) | C(54)-C(55) | 1.393(2) |
| N(2)-C(3) | 1.344(2) | N(4)-C(22) | 1.438(2) | C(37)-C(45) | 1.481(2) | C(55)-C(56) | 1.371(3) |
| C(3)-N(3) | 1.341(2) | N(4)-C(35) | 1.438(2) | N(6)-C(38) | 1.340(2) | N(8)-C(57) | 1.433(2) |
| C(3)-C(16) | 1.471(2) | C(22)-C(23) | 1.395(2) | C(38)-N(7) | 1.345(2) | N(8)-C(70) | 1.438(2) |
| C(4)-C(9) | 1.387(3) | C(22)-C(27) | 1.398(2) | C(38)-C(51) | 1.458(3) | C(57)-C(58) | 1.388(3) |
| C(4)-C(5) | 1.388(2) | C(23)-C(24) | 1.373(2) | C(39)-C(40) | 1.383(3) | C(57)-C(62) | 1.390(3) |
| C(5)-C(6) | 1.378(3) | C(24)-C(25) | 1.379(3) | C(39)-C(44) | 1.386(3) | C(58)-C(59) | 1.380(3) |
| C(6)-C(7) | 1.376(3) | C(25)-C(26) | 1.372(3) | C(40)-C(41) | 1.381(3) | C(59)-C(60) | 1.379(3) |
| C(7)-C(8) | 1.374(3) | C(26)-C(27) | 1.400(2) | C(41)-C(42) | 1.375(3) | C(60)-C(61) | 1.368(3) |
| C(8)-C(9) | 1.381(3) | C(27)-C(28) | 1.513(2) | C(42)-C(43) | 1.365(3) | C(61)-C(62) | 1.398(3) |
| C(10)-C(15) | 1.381(3) | C(28)-C(29) | 1.521(3) | C(43)-C(44) | 1.383(3) | C(62)-C(63) | 1.513(3) |
| C(10)-C(11) | 1.389(3) | C(29)-C(30) | 1.495(2) | C(45)-C(50) | 1.380(3) | C(63)-C(64) | 1.507(3) |
| C(11)-C(12) | 1.384(3) | C(30)-C(31) | 1.387(2) | C(45)-C(46) | 1.384(3) | C(64)-C(65) | 1.502(3) |
| C(12)-C(13) | 1.367(3) | C(30)-C(35) | 1.389(2) | C(46)-C(47) | 1.377(3) | C(65)-C(70) | 1.384(3) |
| C(13)-C(14) | 1.374(3) | C(31)-C(32) | 1.375(3) | C(47)-C(48) | 1.363(3) | C(65)-C(66) | 1.387(3) |
| C(14)-C(15) | 1.394(3) | C(32)-C(33) | 1.381(3) | C(48)-C(49) | 1.376(3) | C(66)-C(67) | 1.378(3) |
| C(16)-C(21) | 1.388(2) | C(33)-C(34) | 1.383(2) | C(49)-C(50) | 1.378(3) | C(67)-C(68) | 1.374(3) |
| C(68)-C(69) | 1.386(3) | C(69)-C(70) | 1.376(2) | | | | |

**Supplementary Table 6. Selected bond angles (°) for AZP-TRZ**

| bond angles (°) | | | | | |
|---|---|---|---|---|---|
| N(1)-C(1)-N(3) | 124.86(17) | C(11)-C(10)-C(2) | 119.94(18) | C(24)-C(23)-C(22) | 121.32(18) |
| N(1)-C(1)-C(4) | 117.97(16) | C(12)-C(11)-C(10) | 120.4(2) | C(23)-C(24)-C(25) | 119.44(18) |
| N(3)-C(1)-C(4) | 117.10(15) | C(13)-C(12)-C(11) | 120.2(2) | C(26)-C(25)-C(24) | 119.52(18) |
| C(1)-N(1)-C(2) | 115.33(16) | C(12)-C(13)-C(14) | 120.2(2) | C(25)-C(26)-C(27) | 122.76(18) |
| N(1)-C(2)-N(2) | 124.89(16) | C(13)-C(14)-C(15) | 120.2(2) | C(22)-C(27)-C(26) | 116.85(16) |
| N(1)-C(2)-C(10) | 117.18(17) | C(10)-C(15)-C(14) | 120.0(2) | C(22)-C(27)-C(28) | 126.81(16) |
| N(2)-C(2)-C(10) | 117.88(17) | C(21)-C(16)-C(17) | 117.03(16) | C(26)-C(27)-C(28) | 116.33(16) |
| C(2)-N(2)-C(3) | 114.88(16) | C(21)-C(16)-C(3) | 120.66(16) | C(27)-C(28)-C(29) | 116.82(15) |
| N(3)-C(3)-N(2) | 124.67(17) | C(17)-C(16)-C(3) | 122.23(16) | C(30)-C(29)-C(28) | 109.03(15) |
| N(3)-C(3)-C(16) | 116.98(15) | C(18)-C(17)-C(16) | 121.81(16) | C(31)-C(30)-C(35) | 118.24(17) |
| N(2)-C(3)-C(16) | 118.30(16) | C(17)-C(18)-C(19) | 120.96(16) | C(31)-C(30)-C(29) | 122.96(17) |
| C(1)-N(3)-C(3) | 115.23(15) | N(4)-C(19)-C(18) | 122.18(15) | C(35)-C(30)-C(29) | 118.43(15) |
| C(9)-C(4)-C(5) | 119.10(18) | N(4)-C(19)-C(20) | 120.84(15) | C(32)-C(31)-C(30) | 120.95(18) |
| C(9)-C(4)-C(1) | 120.18(17) | C(18)-C(19)-C(20) | 116.95(16) | C(31)-C(32)-C(33) | 120.23(17) |
| C(5)-C(4)-C(1) | 120.64(17) | C(21)-C(20)-C(19) | 121.34(16) | C(32)-C(33)-C(34) | 119.86(18) |
| C(6)-C(5)-C(4) | 120.32(19) | C(20)-C(21)-C(16) | 121.82(17) | C(35)-C(34)-C(33) | 119.54(17) |
| C(7)-C(6)-C(5) | 120.1(2) | C(19)-N(4)-C(22) | 123.25(13) | C(34)-C(35)-C(30) | 121.17(16) |
| C(8)-C(7)-C(6) | 120.1(2) | C(19)-N(4)-C(35) | 120.61(14) | C(34)-C(35)-N(4) | 121.41(16) |
| C(7)-C(8)-C(9) | 120.2(2) | C(22)-N(4)-C(35) | 115.98(13) | C(30)-C(35)-N(4) | 117.39(16) |
| C(8)-C(9)-C(4) | 120.15(19) | C(23)-C(22)-C(27) | 120.08(16) | N(7)-C(36)-N(5) | 124.88(16) |
| C(15)-C(10)-C(11) | 119.08(18) | C(23)-C(22)-N(4) | 117.77(16) | N(7)-C(36)-C(39) | 116.99(16) |
| C(15)-C(10)-C(2) | 120.88(18) | C(27)-C(22)-N(4) | 121.98(15) | N(5)-C(36)-C(39) | 118.12(17) |

| | | | | | |
|---|---|---|---|---|---|
| C(37)-N(5)-C(36) | 114.98(16) | C(46)-C(45)-C(37) | 120.61(17) | C(58)-C(57)-C(62) | 120.81(17) |
| N(5)-C(37)-N(6) | 125.02(16) | C(47)-C(46)-C(45) | 120.41(19) | C(58)-C(57)-N(8) | 117.12(17) |
| N(5)-C(37)-C(45) | 118.01(17) | C(48)-C(47)-C(46) | 120.2(2) | C(62)-C(57)-N(8) | 122.06(16) |
| N(6)-C(37)-C(45) | 116.97(16) | C(47)-C(48)-C(49) | 119.9(2) | C(59)-C(58)-C(57) | 120.7(2) |
| C(37)-N(6)-C(38) | 115.51(15) | C(48)-C(49)-C(50) | 120.3(2) | C(60)-C(59)-C(58) | 119.4(2) |
| N(6)-C(38)-N(7) | 123.91(17) | C(49)-C(50)-C(45) | 120.0(2) | C(61)-C(60)-C(59) | 119.65(19) |
| N(6)-C(38)-C(51) | 118.63(16) | C(56)-C(51)-C(52) | 117.48(17) | C(60)-C(61)-C(62) | 122.7(2) |
| N(7)-C(38)-C(51) | 117.43(16) | C(56)-C(51)-C(38) | 120.17(16) | C(57)-C(62)-C(61) | 116.81(18) |
| C(36)-N(7)-C(38) | 115.69(15) | C(52)-C(51)-C(38) | 122.20(16) | C(57)-C(62)-C(63) | 126.26(17) |
| C(40)-C(39)-C(44) | 119.09(18) | C(53)-C(52)-C(51) | 121.67(17) | C(61)-C(62)-C(63) | 116.91(18) |
| C(40)-C(39)-C(36) | 120.21(18) | C(52)-C(53)-C(54) | 120.51(17) | C(64)-C(63)-C(62) | 119.67(18) |
| C(44)-C(39)-C(36) | 120.70(18) | C(53)-C(54)-N(8) | 122.16(16) | C(65)-C(64)-C(63) | 112.92(18) |
| C(41)-C(40)-C(39) | 120.3(2) | C(53)-C(54)-C(55) | 117.84(17) | C(70)-C(65)-C(66) | 117.91(18) |
| C(42)-C(41)-C(40) | 120.0(2) | N(8)-C(54)-C(55) | 119.98(16) | C(70)-C(65)-C(64) | 118.60(17) |
| C(43)-C(42)-C(41) | 120.2(2) | C(56)-C(55)-C(54) | 120.82(17) | C(66)-C(65)-C(64) | 123.49(18) |
| C(42)-C(43)-C(44) | 120.2(2) | C(55)-C(56)-C(51) | 121.61(17) | C(67)-C(66)-C(65) | 121.30(19) |
| C(43)-C(44)-C(39) | 120.2(2) | C(54)-N(8)-C(57) | 120.87(14) | C(68)-C(67)-C(66) | 119.96(18) |
| C(50)-C(45)-C(46) | 119.08(18) | C(54)-N(8)-C(70) | 118.67(14) | C(67)-C(68)-C(69) | 119.66(18) |
| C(50)-C(45)-C(37) | 120.30(18) | C(57)-N(8)-C(70) | 117.34(14) | C(70)-C(69)-C(68) | 119.90(18) |

| | | | | | |
|---|---|---|---|---|---|
| C(69)-C(70)-C(65) | 121.26(17) | C(69)-C(70)-N(8) | 119.70(17) | C(65)-C(70)-N(8) | 118.98(16) |

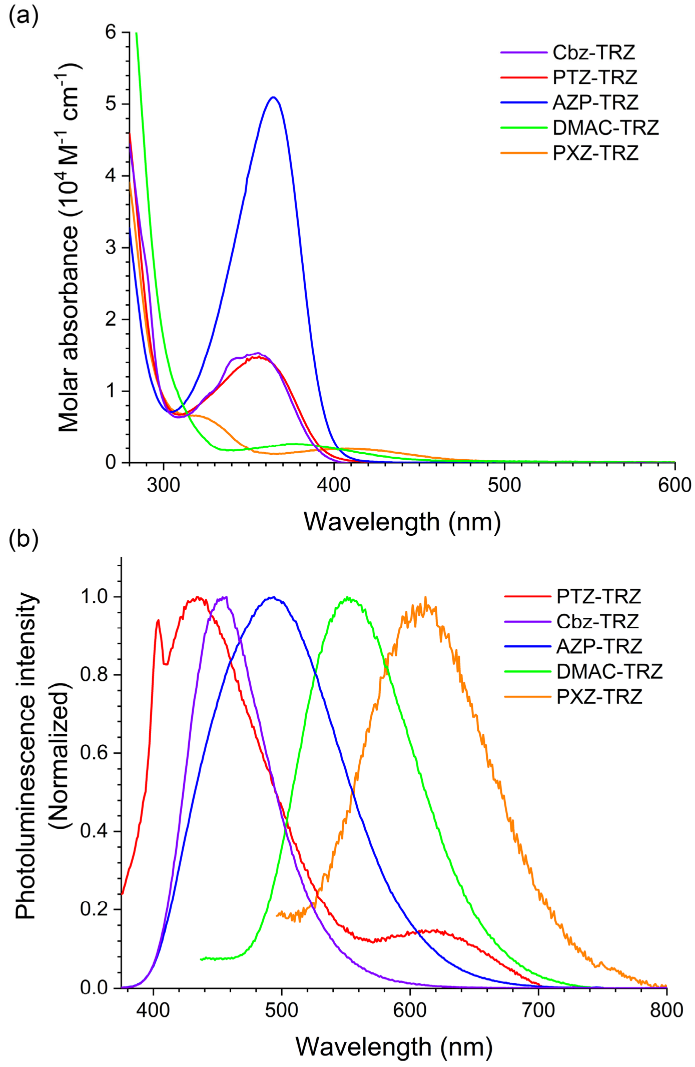


**Supplementary Figure 1. Steady-state electronic spectra**. (a) UV–vis absorption and (b) photoluminescence spectra of EtOAc solutions containing 50 μM compounds (298 K). Excitation wavelengths: Cbz-TRZ, 363 nm; AZP-TRZ, 372 nm; DMAC-TRZ, 379 nm; PXZ-TRZ, 414 nm; PTZ-TRZ, 359 nm.

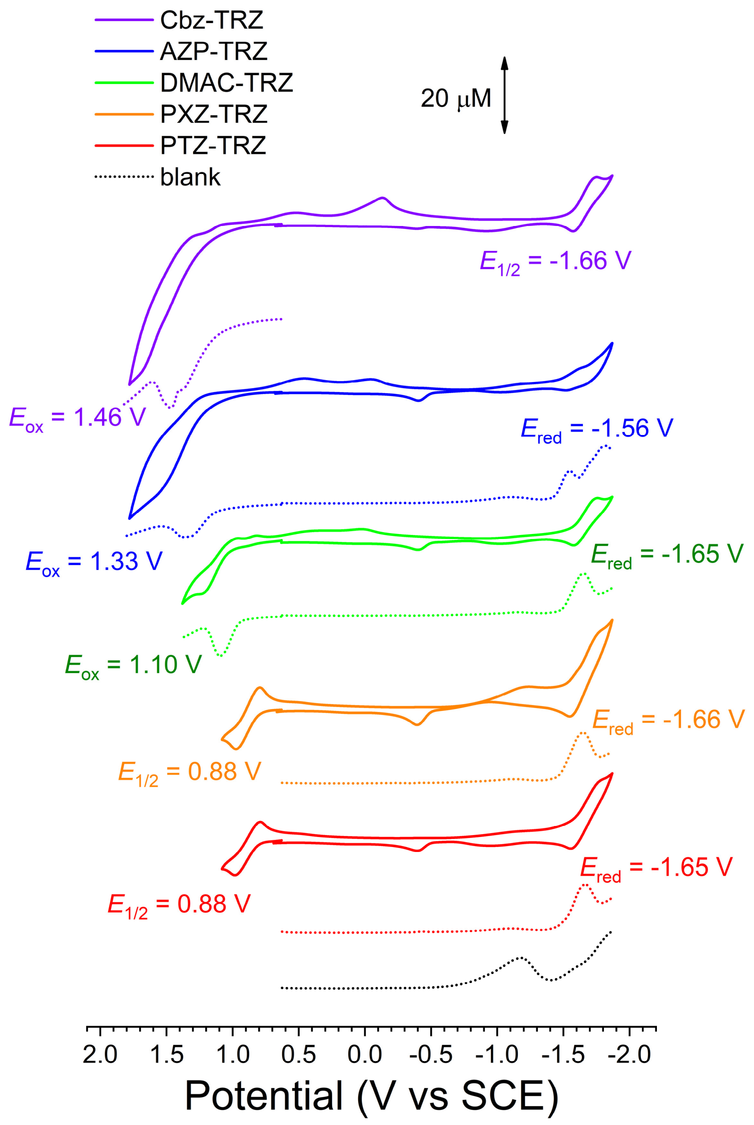


**Supplementary Figure 2. Determination of redox potentials.** Cyclic (CV, solid lines) and differential pulse (DPV, dotted lines) voltammograms for Ar-saturated THF containing 0.10 M $TBAPF_6$ and 2.0 mM compound. A Pt microdisc and a Pt wire were employed as the working and counter electrodes, respectively. An $Ag/AgNO_3$ couple served as the pseudo reference electrode. The potentials were corrected by using the $Fc/Fc^+$ external standard. Scan rates = 0.10 V $s^{-1}$ (CV) and 4 mV $s^{-1}$ (DPV). The voltammogram of a blank (i.e., Ar-saturated THF containing 0.10 M $TBAPF_6$) is included (dotted black line).

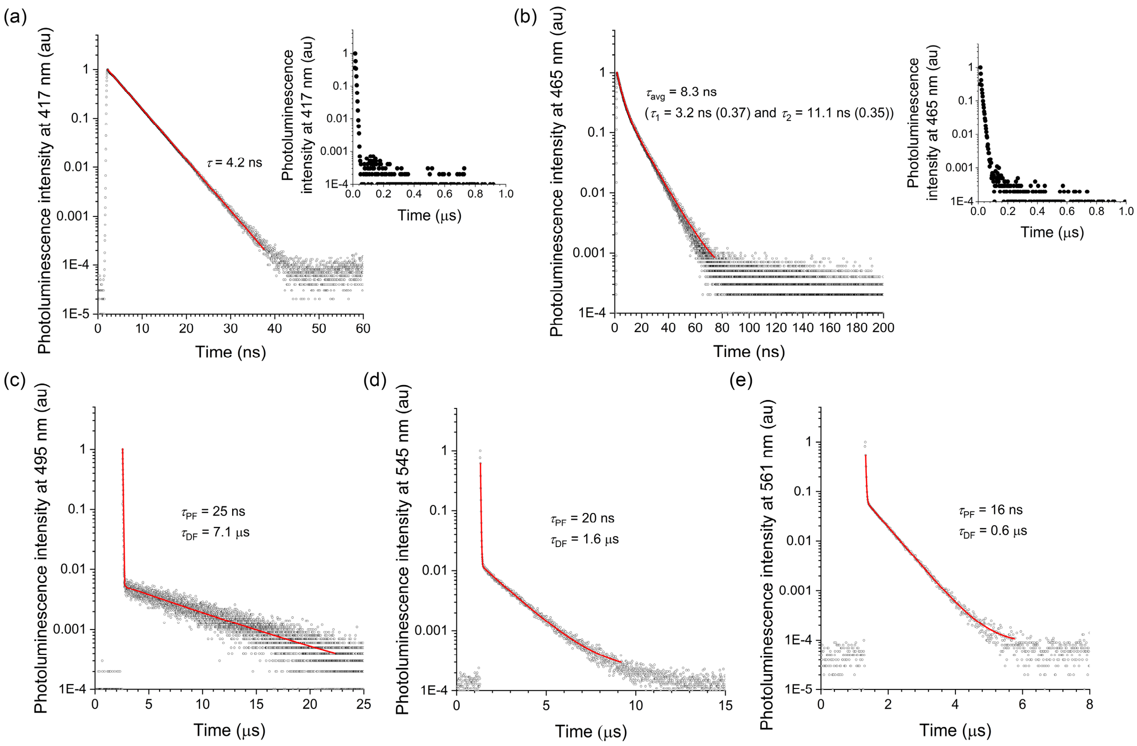


**Supplementary Figure 3. Determination of fluorescence lifetime.** Photoluminescence decay traces of (a) Cbz-TRZ, (b) AZP-TRZ, (c) DMAC-TRZ, (d) PXZ-TRZ, and (e) PTZ-TRZ recorded after picosecond pulsed laser excitation at 377 nm. Observation wavelengths: Cbz-TRZ, 417 nm; AZP-TRZ, 465 nm; DMAC-TRZ, 495 nm; PXZ-TRZ, 545 nm; PTZ-TRZ, 561 nm. The burst mode was employed to monitor the traces in (c–e) and those in the inset graphs in (a,b), whereas the normal mode was used to collect the traces in (a,b). The 10 μM sample was dissolved in Ar-saturated toluene. The red curves are fits to mono- (a) and biexponential (b–e) decay models.

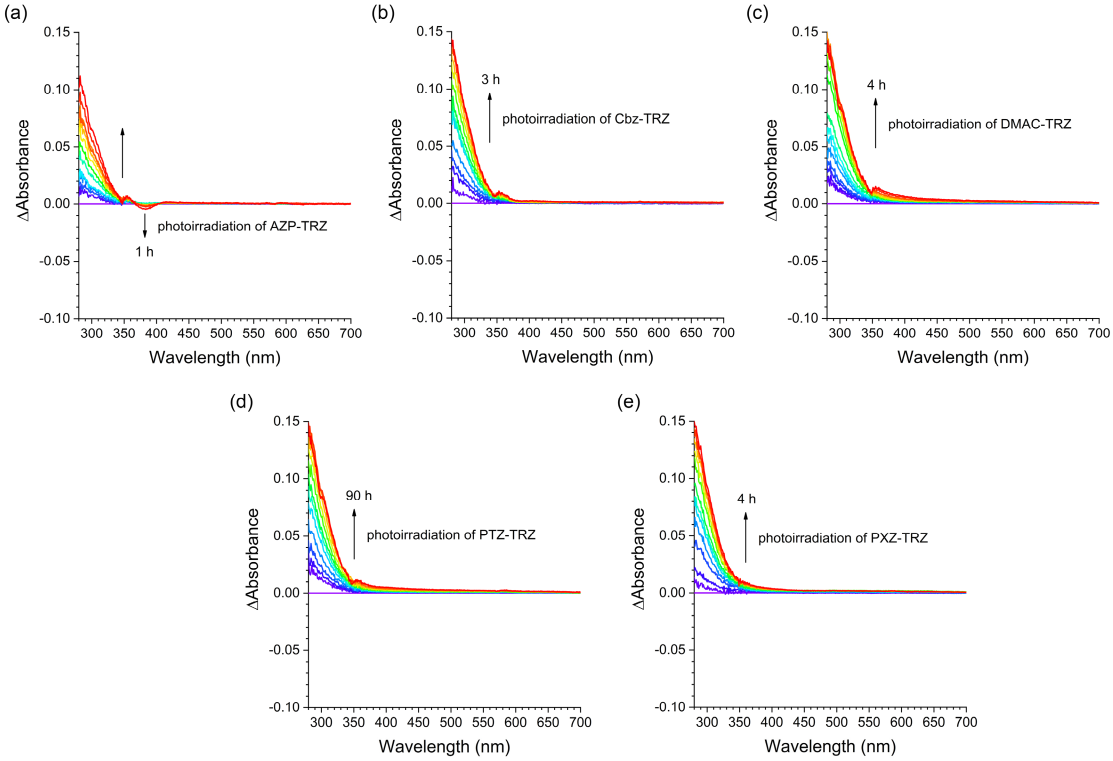


**Supplementary Figure 4. Photolytic degradation of films.** UV–vis absorption difference spectra of thick mCBP films containing 10 wt % dopants recorded under photoillumination of broad-band light from a Xe lamp (300 W): (a) AZP-TRZ, (b) Cbz-TRZ, (c) DMAC-TRZ, (d) PTZ-TRZ, and (e) PXZ-TRZ.

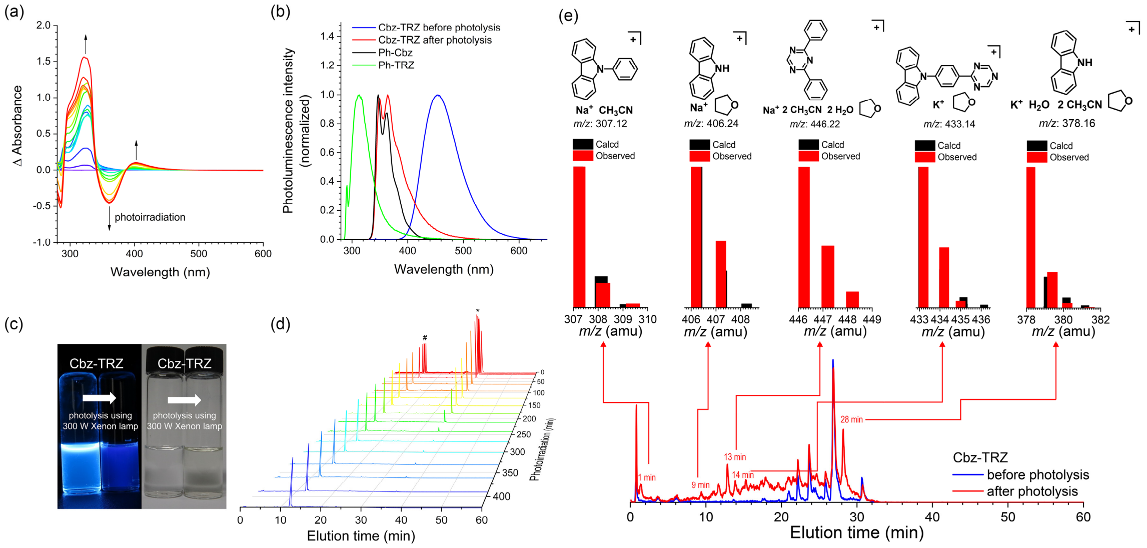


**Supplementary Figure 5. Intrinsic degradation of Cbz-TRZ**. (a) Changes in the UV–vis absorption spectra upon continuous photolysis of Ar-saturated THF containing 100 μM Cbz-TRZ under illumination of broad-band light from a Xe lamp (300 W) for 180 min. (b) Comparison of the photoluminescence spectra of the 100 μM Cbz-TRZ solution before (blue) and after (red) photolysis, and of the fragment models (100 μM in THF), Ph-Cbz (black) and Ph-TRZ (green). Excitation wavelength = 305 nm (Cbz-TRZ) and 295 nm (Ph-Cbz and Ph-TRZ). Note that the photoluminescence spectra of Cbz-TRZ obtained using a photoexcitation wavelength of 295 nm were identical to those shown in (b). (c) Photo showing the photoluminescence and absorption changes of Cbz-TRZ after photolysis. (d) Liquid chromatograms (VWD) of an Ar-saturated THF/$CH_3CN$ solution containing 100 μM Cbz-TRZ taken at various photolysis times. The peaks marked with # and * correspond to the benzophenone internal standard (200 μM) and Cbz-TRZ, respectively. The benzophenone internal standard was added after the photolysis. (e) An overlay of the chromatograms (MSD) of Cbz-TRZ before (blue) and after (red) photolysis. The mass spectra shown on top correspond to the calculated (black) and observed (red) isotope distributions of the major peak of the indicated chromatographic peaks. The chemical structures above the mass spectra are the proposed structures corresponding the calculated values.

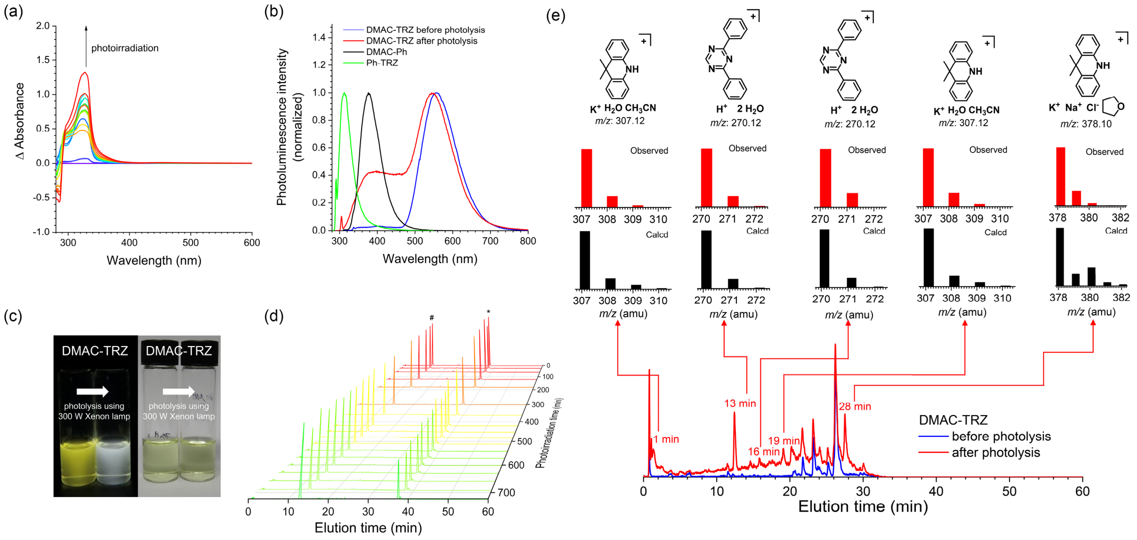


**Supplementary Figure 6. Intrinsic degradation of DMAC-TRZ**. (a) Changes in the UV–vis absorption spectra upon continuous photolysis of Ar-saturated THF containing 100 μM DMAC-TRZ under illumination of broad-band light from a Xe lamp (300 W) for 180 min. (b) Comparison of the photoluminescence spectra of the 100 μM DMAC-TRZ solution before (blue) and after (red) photolysis, and of the fragment models (100 μM in THF), DMAC-Ph (black) and Ph-TRZ (green). Excitation wavelength = 305 nm (DMAC-TRZ) and 295 nm (DMAC-Ph and Ph-TRZ). Note that the photoluminescence spectra of DMAC-TRZ obtained using a photoexcitation wavelength of 295 nm were identical to those shown in (b). (c) Photo showing the photoluminescence and absorption changes of DMAC-TRZ after photolysis. (d) Liquid chromatograms (VWD) of an Ar-saturated THF/$CH_3CN$ solution containing 100 μM DMAC-TRZ taken at various photolysis times. The peaks marked with # and * correspond to the benzophenone internal standard (200 μM) and DMAC-TRZ, respectively. The benzophenone internal standard was added after the photolysis. (e) An overlay of the chromatograms (MSD) of DMAC-TRZ before (blue) and after (red) photolysis. The mass spectra shown on top correspond to the calculated (black) and observed (red) isotope distributions of the major peak of the indicated chromatographic peaks. The chemical structures above the mass spectra are the proposed structures corresponding the calculated values.

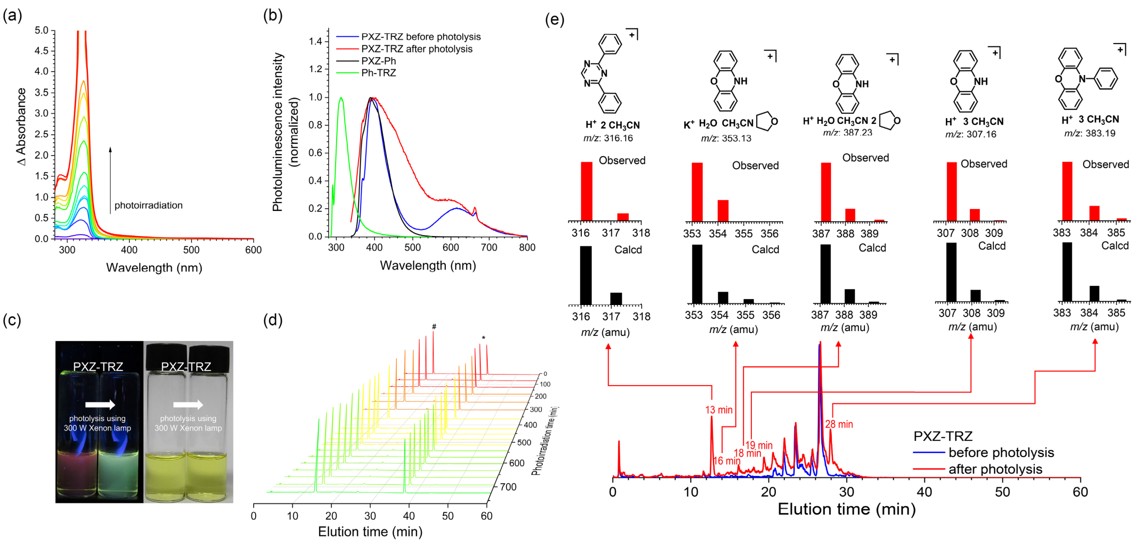


**Supplementary Figure 7. Intrinsic degradation of PXZ-TRZ.** (a) Changes in the UV–vis absorption spectra upon continuous photolysis of Ar-saturated THF containing 100 μM PXZ-TRZ under illumination of broad-band light from a Xe lamp (300 W) for 180 min. (b) Comparison of the photoluminescence spectra of the 100 μM PXZ-TRZ solution before (blue) and after (red) photolysis, and of the fragment models (100 μM in THF), PXZ-Ph (black) and Ph-TRZ (green). Excitation wavelength = 331 nm (PXZ-TRZ) and 288 nm (PXZ-Ph and Ph-TRZ). Note that the photoluminescence spectra of PXZ-TRZ obtained using a photoexcitation wavelength of 288 nm were identical to those shown in (b). (c) Photo showing the photoluminescence and absorption changes of PXZ-TRZ after photolysis. (d) Liquid chromatograms (VWD) of an Ar-saturated THF/$CH_3CN$ solution containing 100 μM PXZ-TRZ taken at various photolysis times. The peaks marked with # and * correspond to the benzophenone internal standard (200 μM) and PXZ-TRZ, respectively. The benzophenone internal standard was added after the photolysis. (e) An overlay of the chromatograms (MSD) of PXZ-TRZ before (blue) and after (red) photolysis. The mass spectra shown on top correspond to the calculated (black) and observed (red) isotope distributions of the major peak of the indicated chromatographic peaks. The chemical structures above the mass spectra are the proposed structures corresponding the calculated values.

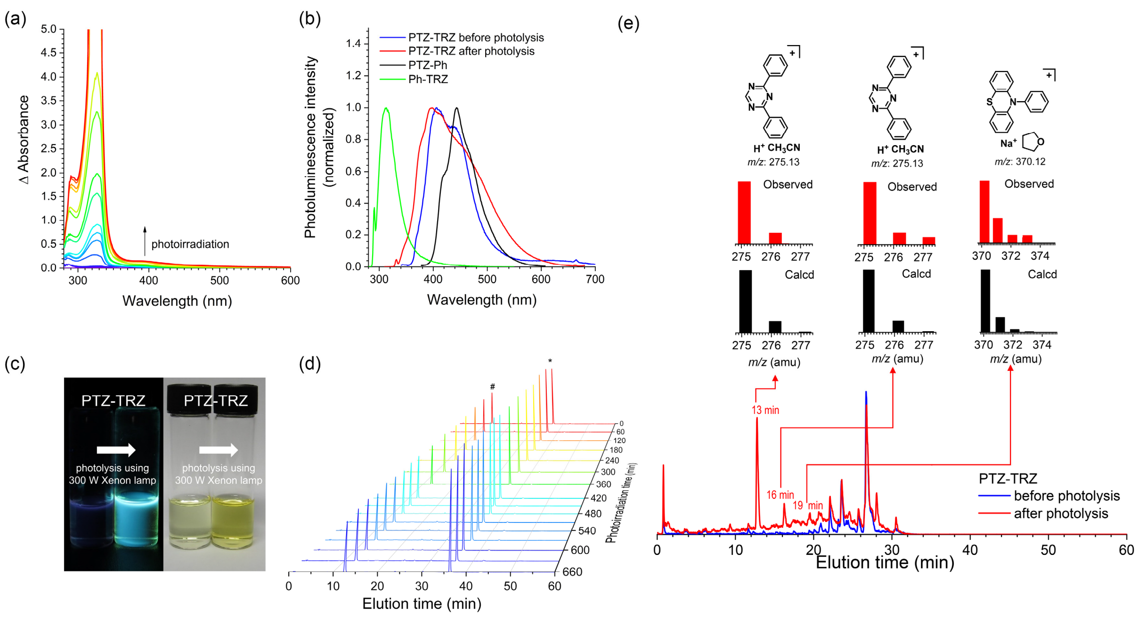


**Supplementary Figure 8. Intrinsic degradation of PTZ-TRZ.** (a) Changes in the UV–vis absorption spectra upon continuous photolysis of Ar-saturated THF containing 100 μM PTZ-TRZ under illumination of broad-band light from a Xe lamp (300 W) for 180 min. (b) Comparison of the photoluminescence spectra of the 100 μM PTZ-TRZ solution before (blue) and after (red) photolysis, and of the fragment models (100 μM in THF), PTZ-Ph (black) and Ph-TRZ (green). Excitation wavelength = 331 nm (PTZ-TRZ) and 288 nm (PTZ-Ph and Ph-TRZ). Note that the photoluminescence spectra of PTZ-TRZ obtained using a photoexcitation wavelength of 288 nm were identical to those shown in (b). (c) Photo showing the photoluminescence and absorption changes of PTZ-TRZ after photolysis. (d) Liquid chromatograms (VWD) of an Ar-saturated THF/$CH_3CN$ solution containing 100 μM PTZ-TRZ taken at various photolysis times. The peaks marked with # and * correspond to the benzophenone internal standard (200 μM) and PTZ-TRZ, respectively. The benzophenone internal standard was added after the photolysis. (e) An overlay of the chromatograms (MSD) of PTZ-TRZ before (blue) and after (red) photolysis. The mass spectra shown on top correspond to the calculated (black) and observed (red) isotope distributions of the major peak of the indicated chromatographic peaks. The chemical structures above the mass spectra are the proposed structures corresponding the calculated values.

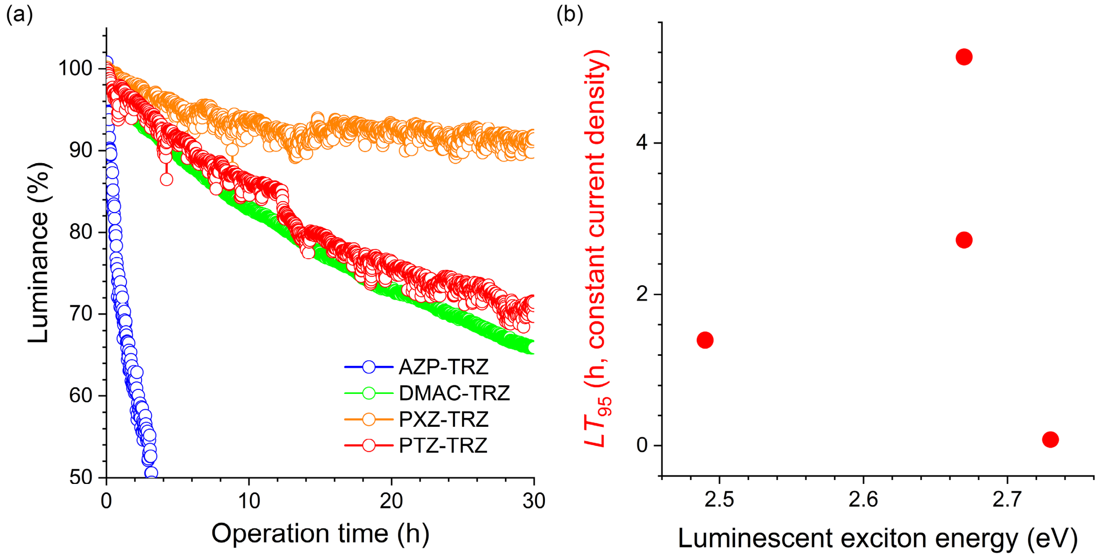


**Supplementary Figure 9. Operation lifetime.** (a) Changes in the % luminance during operation of the devices driven at a current density of 9.25 mA $cm^{-2}$. (b) Correlation of $LT_{95}$ with the luminescent exciton energy determined from the electroluminescence spectra.

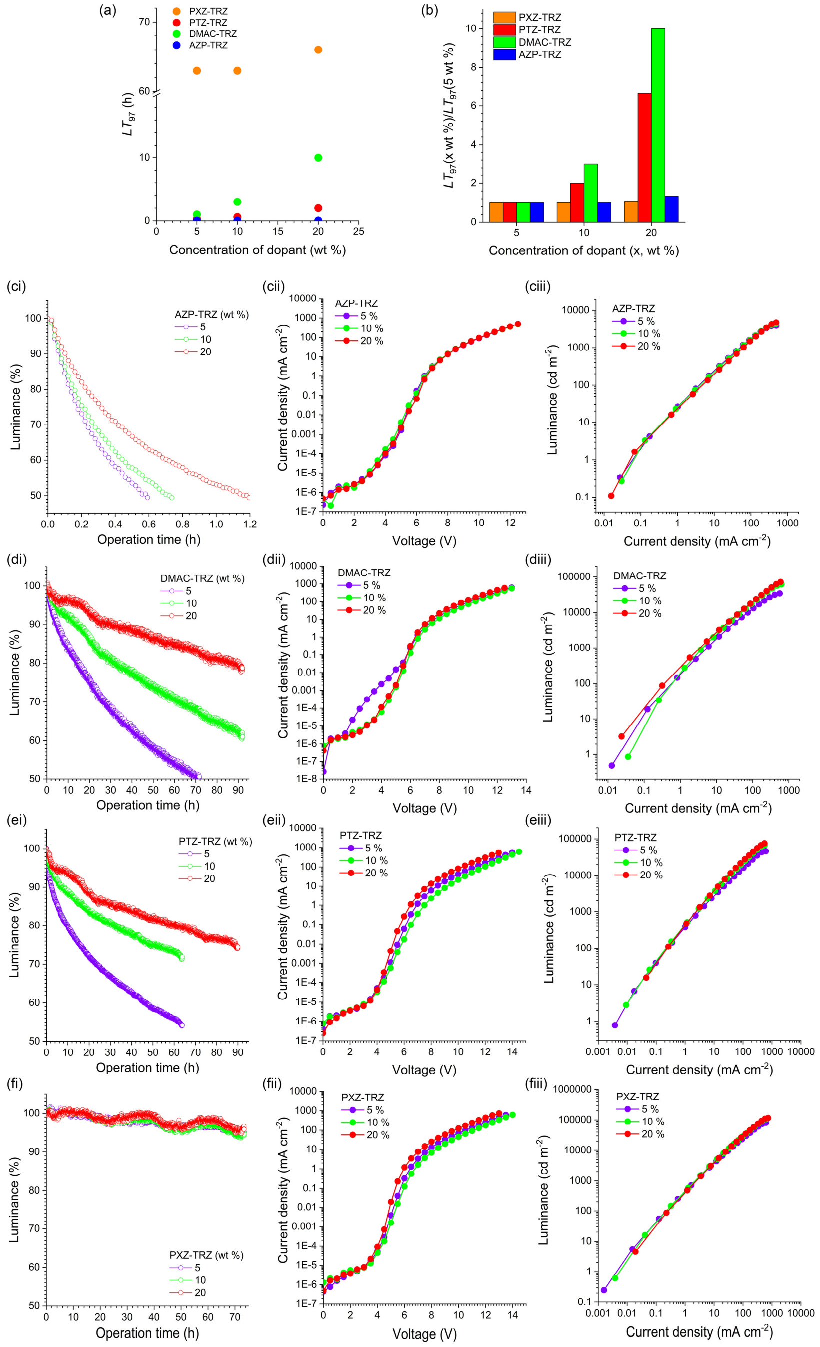


**Supplementary Figure 10. Concentration dependence.** (a) $LT_{97}$ of the devices with emitting layers of 5, 10, and 20 wt % dopant concentrations. (b) Relative increases in $LT_{97}$ of the devices relative to that of 5 wt % device. (c–f) Plots of luminance decays as functions of operation time (i),

current densities as functions of applied voltage (ii), and % luminance as functions of current density (iii) of the devices having 5, 10, and 20 wt % of dopants: c, AZP-TRZ; d, DMAC-TRZ; e, PTZ-TRZ; f, PXZ-TRZ. Changes in the % luminance were recorded during operation of the devices driven at following constant current densities defined at an initial luminance of 200 cd $m^{-2}$: AZP-TRZ, 0.033 mA $cm^{-2}$ (5 wt %), 0.036 mA $cm^{-2}$ (10 wt %), and 0.042 mA $cm^{-2}$ (20 wt %); DMAC-TRZ, 0.040 mA $cm^{-2}$ (5 wt %), 0.032 mA $cm^{-2}$ (10 wt %), and 0.027 mA $cm^{-2}$ (20 wt %); PXZ-TRZ, 0.017 mA $cm^{-2}$ (5 wt %), 0.018 mA $cm^{-2}$ (10 wt %), and 0.019 mA $cm^{-2}$ (20 wt %); PTZ-TRZ, 0.022 mA $cm^{-2}$ (5 wt %), 0.018 mA $cm^{-2}$ (10 wt %), and 0.018 mA $cm^{-2}$ (20 wt %).

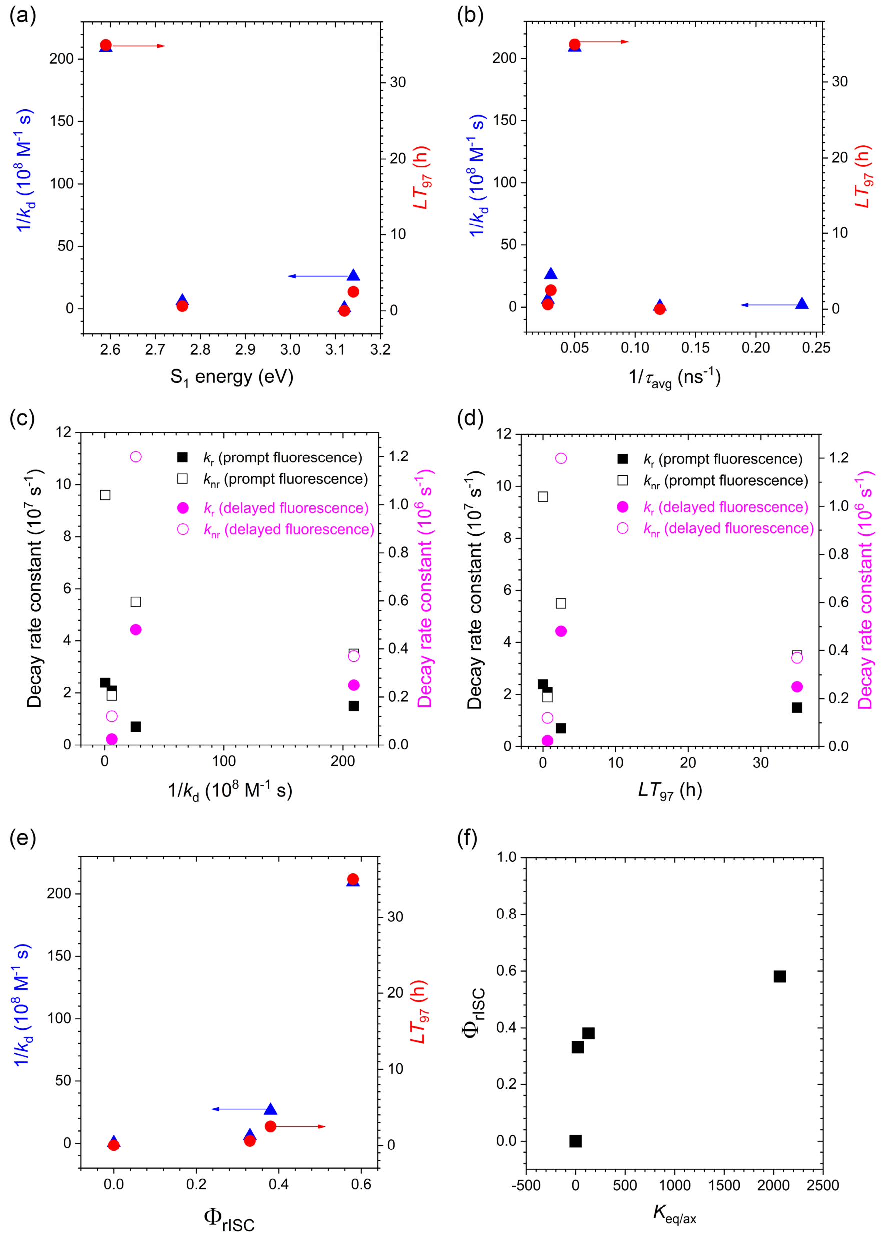


**Supplementary Figure 11. Correlating stability and molecular factors.** (a) Plots of the intrinsic stability ($1/k_d$, blue triangles) and the operational lifetime ($LT_{97}$, red circles) as a function of the singlet excited state ($S_1$) energies of the compounds. (b) Plots of the intrinsic stability ($1/k_d$, blue triangles) and the operational lifetime ($LT_{97}$, red circles) as a function of the natural decay rate

($1/\tau_{avg}$) of the compounds. (c) Plots of the radiative ($k_r$) and non-radiative ($k_{nr}$) rate constants for prompt (black symbols) and delayed fluorescence (pink symbols) as a function of $1/k_d$. (d) Plots of the radiative ($k_r$) and non-radiative ($k_{nr}$) rate constants for prompt (black symbols) and delayed fluorescence (pink symbols) as a function of $LT_{97}$. (e) Plots of the intrinsic stability ($1/k_d$, blue triangles) and the operational lifetime ($LT_{97}$, red circles) as a function of the quantum yield for rISC ($\Phi_{rISC}$). (f) Plot of $\Phi_{rISC}$ as a function of the Boltzmann factor of the pseudo-equatorial form and the pseudo-axial form ($K_{eq/ax}$).

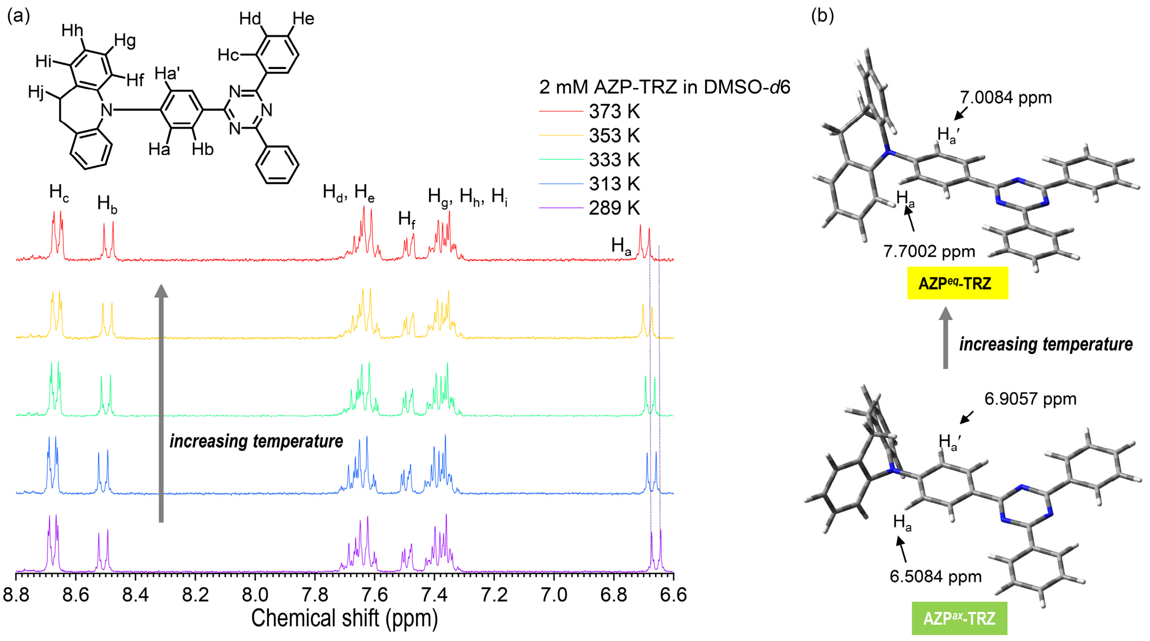


**Supplementary Figure 12. Conformeric change of AZP-TRZ.** (a) Variable-temperature (373–289 K) $^{1}H$ NMR (300 MHz, DMSO-$d_6$) spectra of 2.0 mM AZP-TRZ. (b) Optimized geometries of the simulated (CAM–B3LYP/6–311+G(d,p); GIAO) $^{1}H$ NMR chemical shifts of the $H_a$ and $H_a'$ in the quasi-axial (AZP$^{ax}$-TRZ) and the quasi-equatorial (AZP$^{eq}$-TRZ) forms.

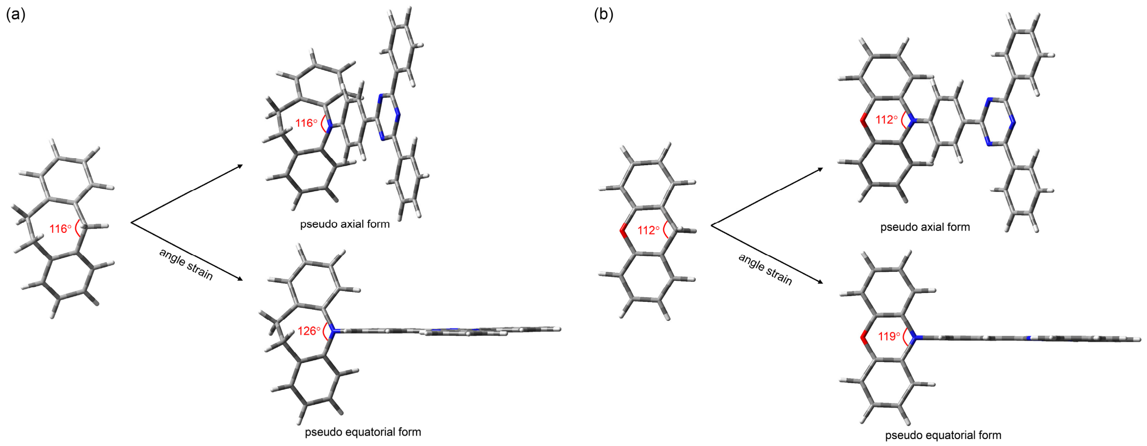


**Supplementary Figure 13. Ring strain.** Comparisons of the C–N–C angle (labeled in red) in the cyclic structures of the model compound (left) and the pseudo-axial (top right) and the pseudo-equatorial (top bottom) forms of (a) AZP-TRZ and (b) PXZ-TRZ. The structure of the model compound involved a $sp^3$-hybridized carbon in place of a nitrogen atom. All the structures were fully optimized at the CAM-B3LYP level of theory employing the 6–311+G(d,p) functional. The greater C–N–C angles indicate the presence of more angle strain in the pseudo-equatorial forms.

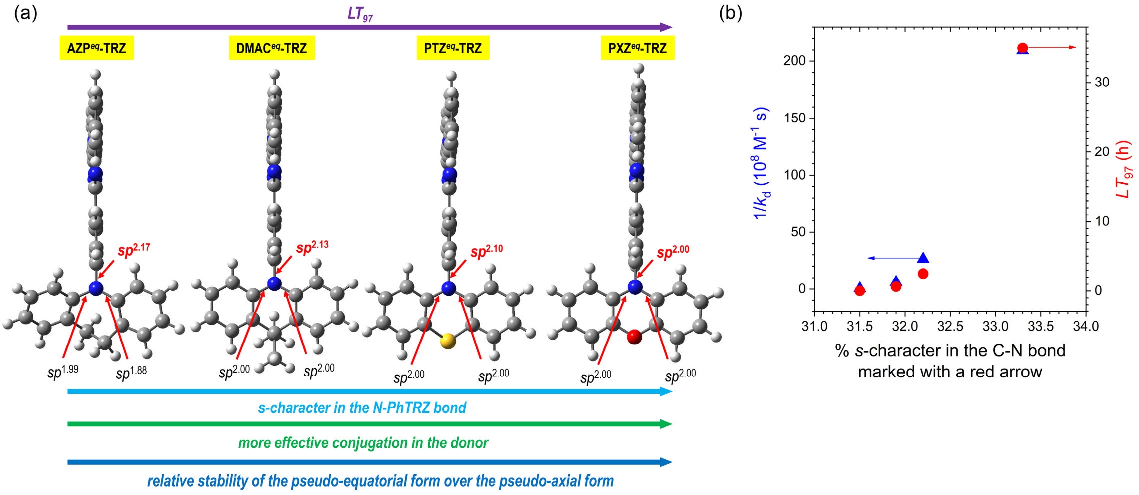


**Supplementary Figure 14. *s*-character in pseudo-equatorial forms**. (a) Natural bond orbital analyses (CAM-B3LYP/6–311+G(d,p)) for the three C–N bonds in the pseudo-equatorial forms of the donor–TRZ compounds. The colored horizontal arrows indicate the increasing orders $LT_{97}$ (violet), *s*-character (sky blue), the extent of effective conjugation in the donor (green), and the relative stability of the pseudo-equatorial form over the pseudo-axial form (blue). (b) Correlations of the % *s*-character with the intrinsic stability ($1/k_d$, blue) and the operational lifetime ($LT_{97}$, red). The increased *s*-character strengthens the C–N bond that is prone to excitonic cleavage. In addition, the pseudo-equatorial form becomes more stable at greater *s*-character. The latter effect directly linked to a more population of the pseudo-equatorial form over the pseudo-axial form.

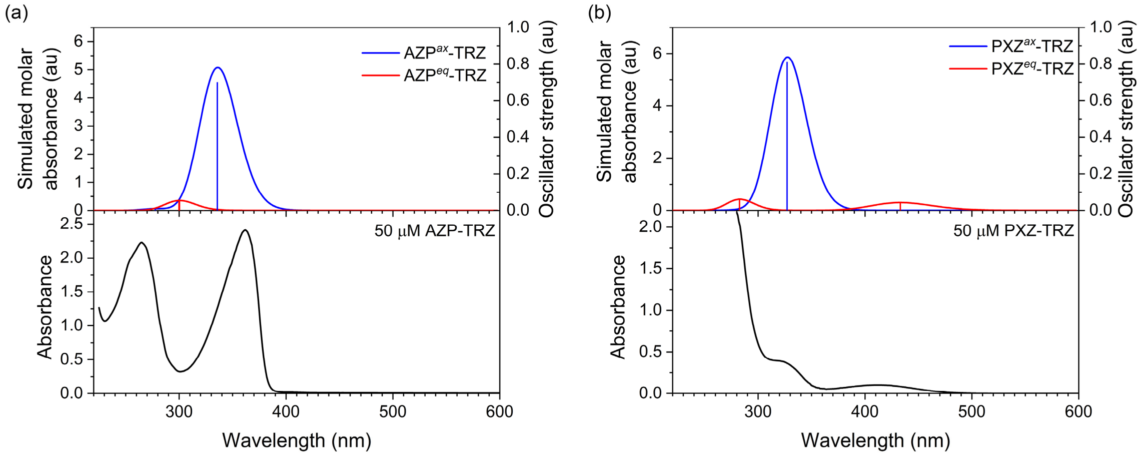


**Supplementary Figure 15. Simulation of the electronic spectra.** Comparisons of the simulated (TD–CAM–B3LYP/6–311+G(d,p), top panels) and experimental (50 μM in THF, 298 K, bottom panels) UV–vis absorption spectra of (a) AZP-TRZ and (b) PXZ-TRZ. The blue and red curves in the top panels correspond to the simulated spectra for the pseudo-axial and equatorial-forms, respectively.

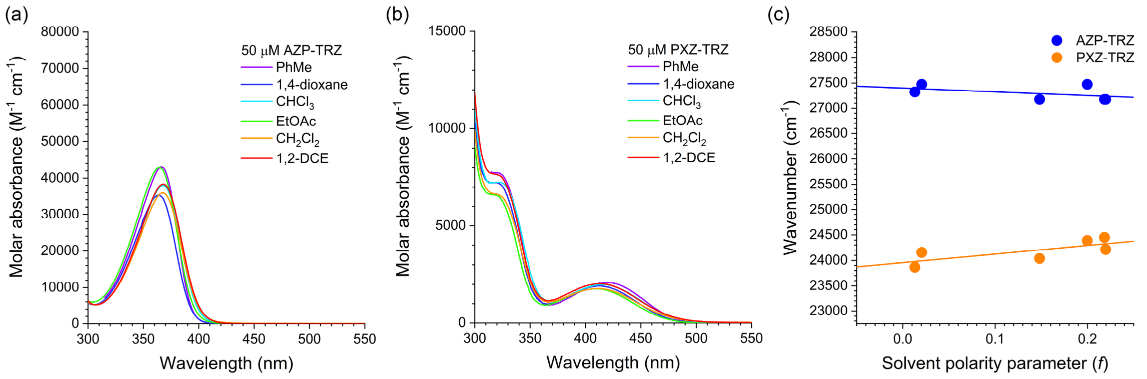


**Supplementary Figure 16. Solvatochromism.** UV–vis absorption spectra of (a) 50 μM AZP-TRZ and (b) 50 μM PXZ-TRZ obtained in various solvents. (c) Lippert–Mataga plot of the absorption peaks of AZP-TRZ (blue) and PXZ-TRZ (orange) as a function of solvent polarity parameter ($f$). $f = (\varepsilon - 1)/(2\varepsilon + 1) - (n^2 - 1)/(2n^2 + 1)$, where $\varepsilon$ and $n$ are the dielectric constant and the refractive index of solvent, respectively. The dipole moments of the conformers of the AZP-TRZ and PXX-TRZ were predicted through CAM–B3LYP/6–311+G(d,p) calculations to be 3.7114 Debye for AZP$^{ax}$-TRZ, 1.1223 Debye for AZP$^{eq}$-TRZ, 2.4284 Debye for PXZ$^{ax}$-TRZ, and 3.0318 Debye for PXZ$^{eq}$-TRZ.

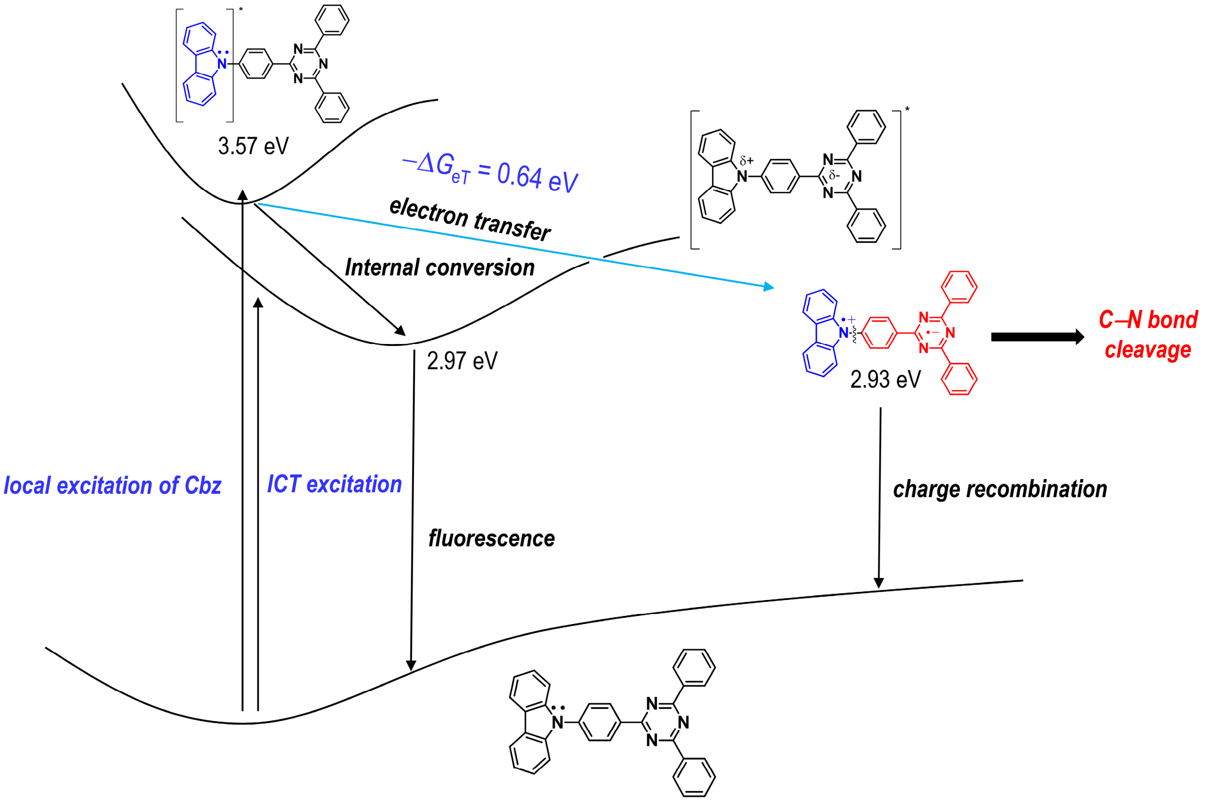


**Supplementary Figure 17. Plausible mechanism for degradation of Cbz-TRZ.** Schematic representation for an alternative degradation pathway of Cbz-TRZ.

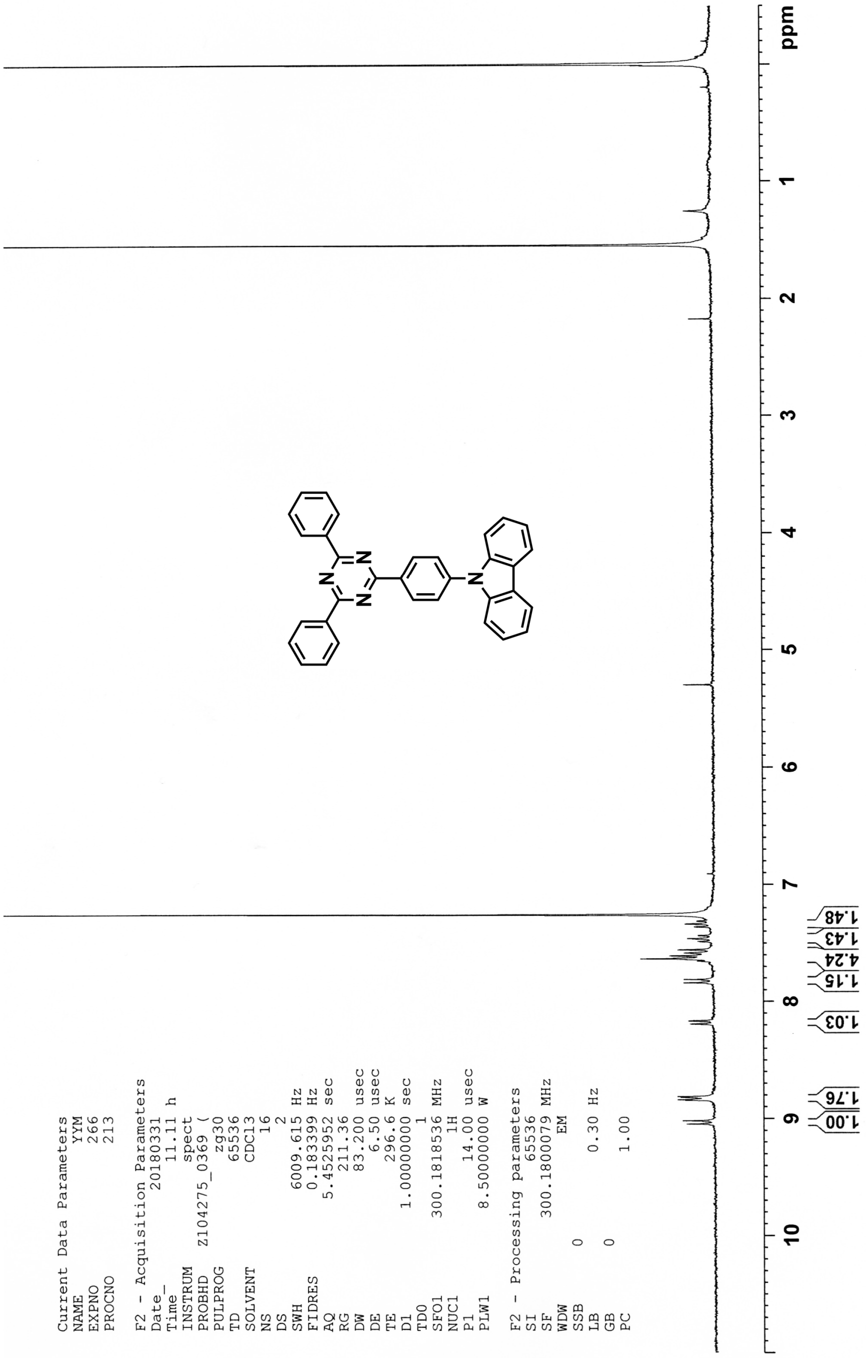


**Supplementary Figure 18. $^1$H NMR (300 MHz, $CDCl_3$) spectrum of Cbz-TRZ.**

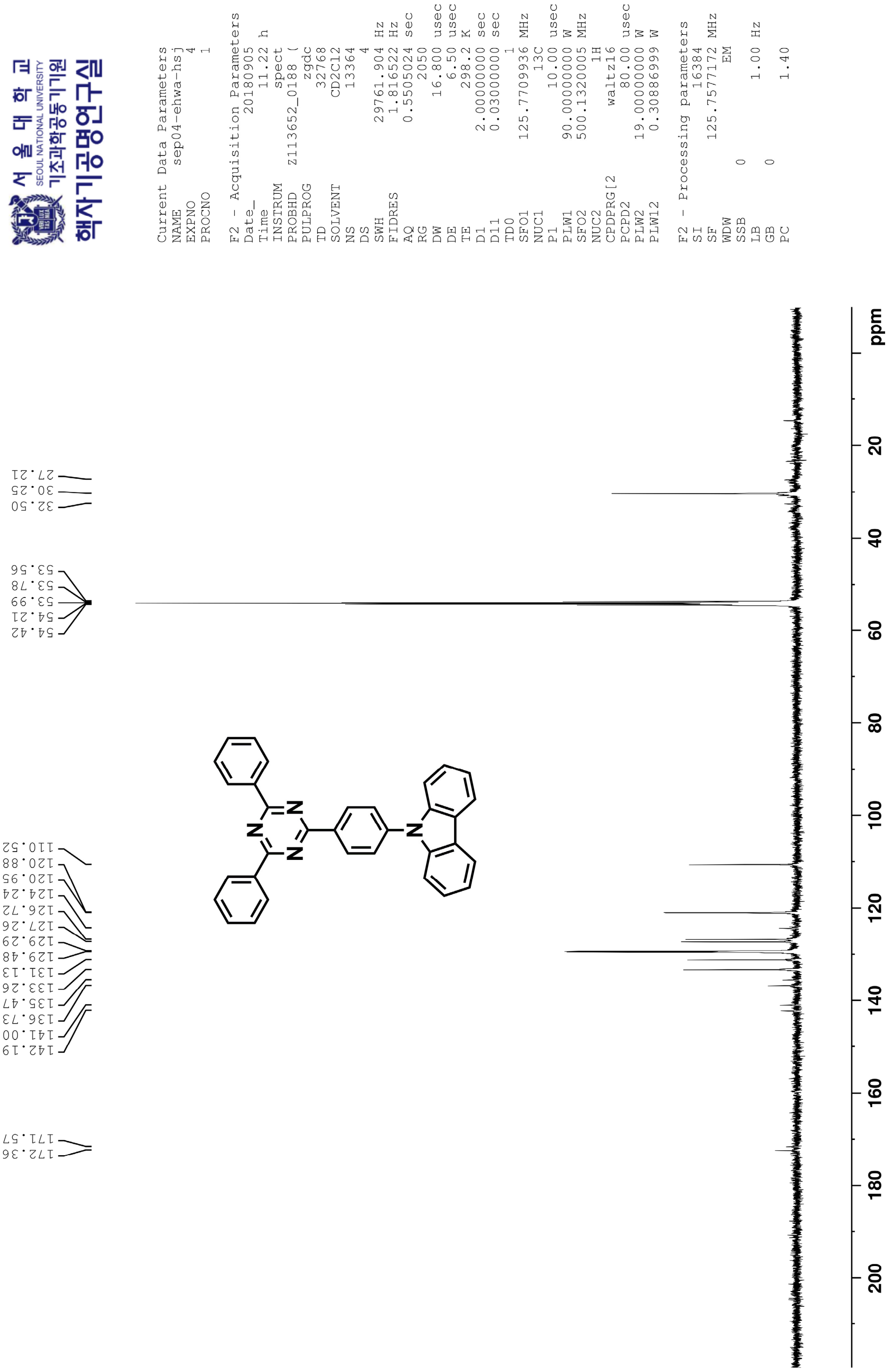


**Supplementary Figure 19.** $^{13}C\{^1H\}$ **NMR (126 MHz,** $CD_2Cl_2$**) spectrum of Cbz-TRZ.**

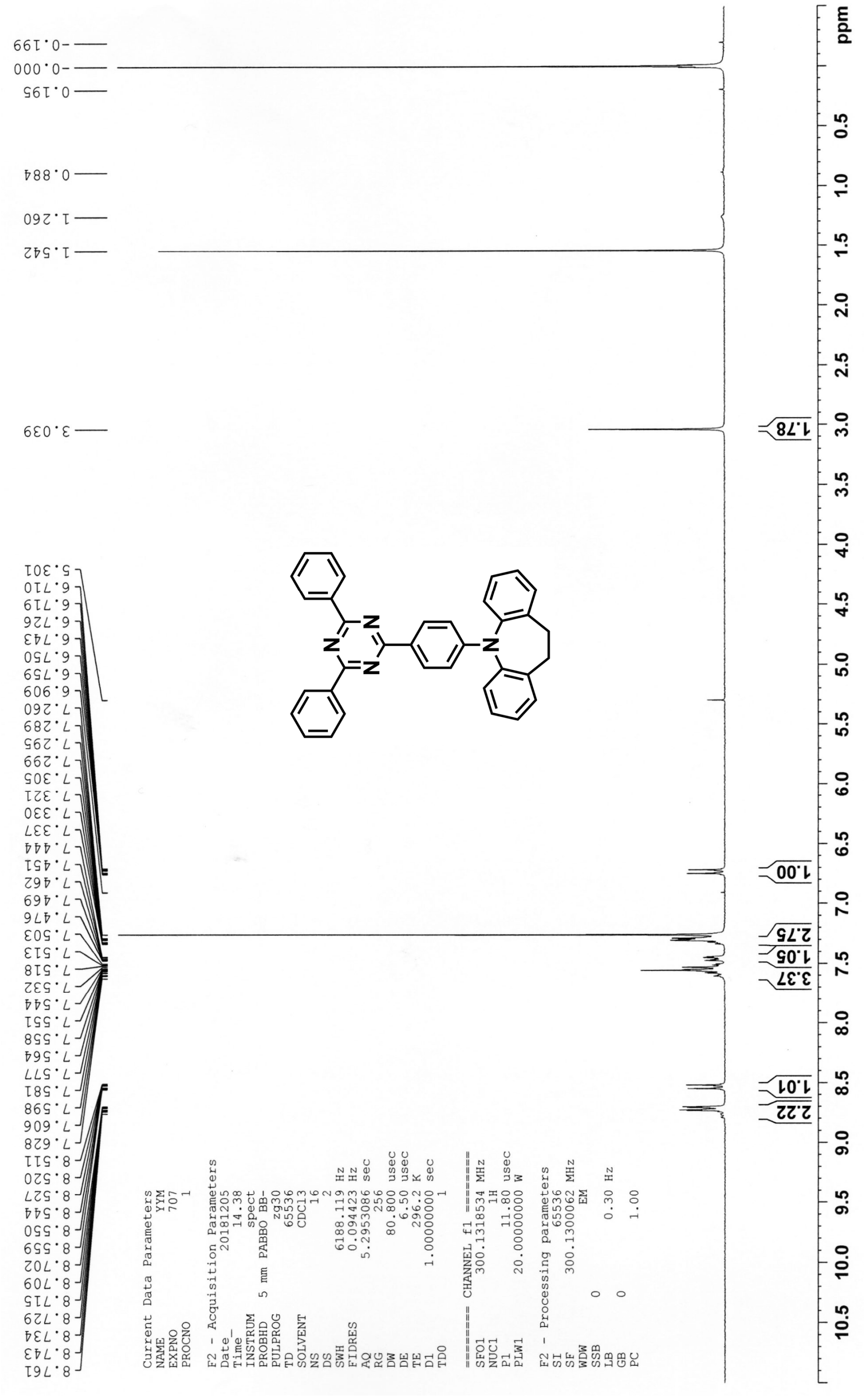

**Supplementary Figure 20. $^{1}$H NMR (300 MHz, $CDCl_3$) spectrum of AZP-TRZ.**

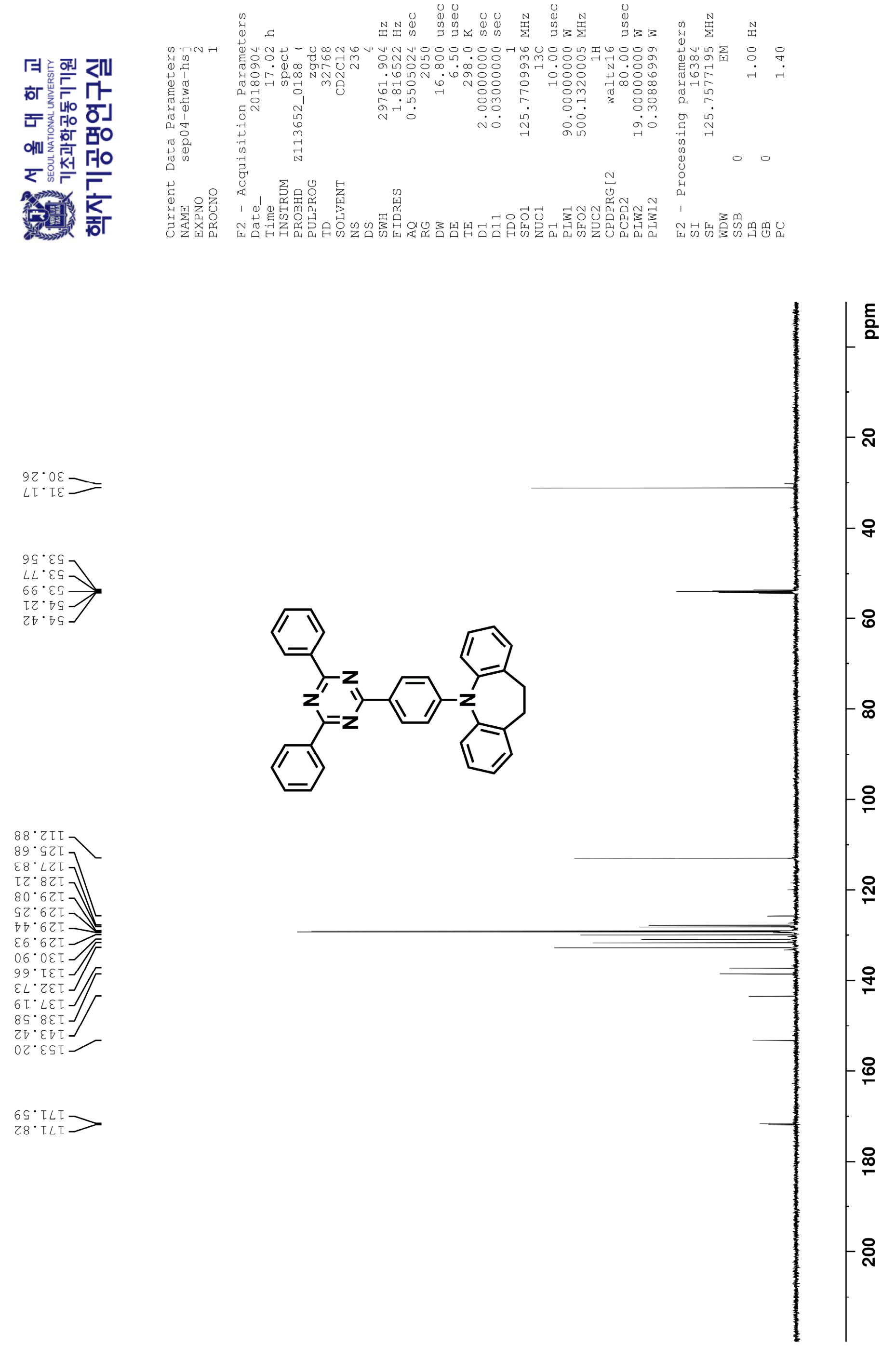

**Supplementary Figure 21.** $^{13}C\{^{1}H\}$ **NMR (126 MHz,** $CD_2Cl_2$**) spectrum of AZP-TRZ.**

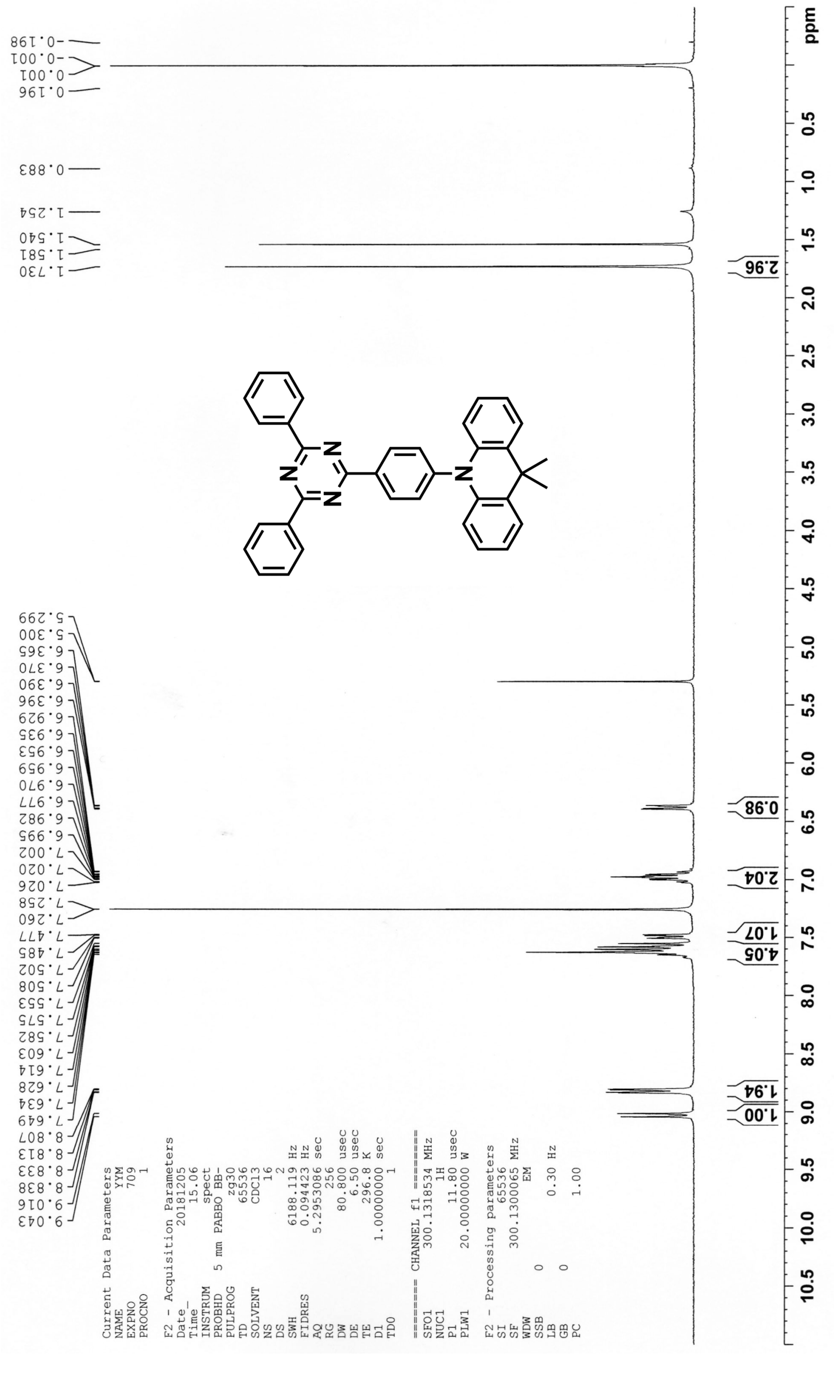

**Supplementary Figure 22. $^1$H NMR (300 MHz, $CDCl_3$) spectrum of DMAC-TRZ.**

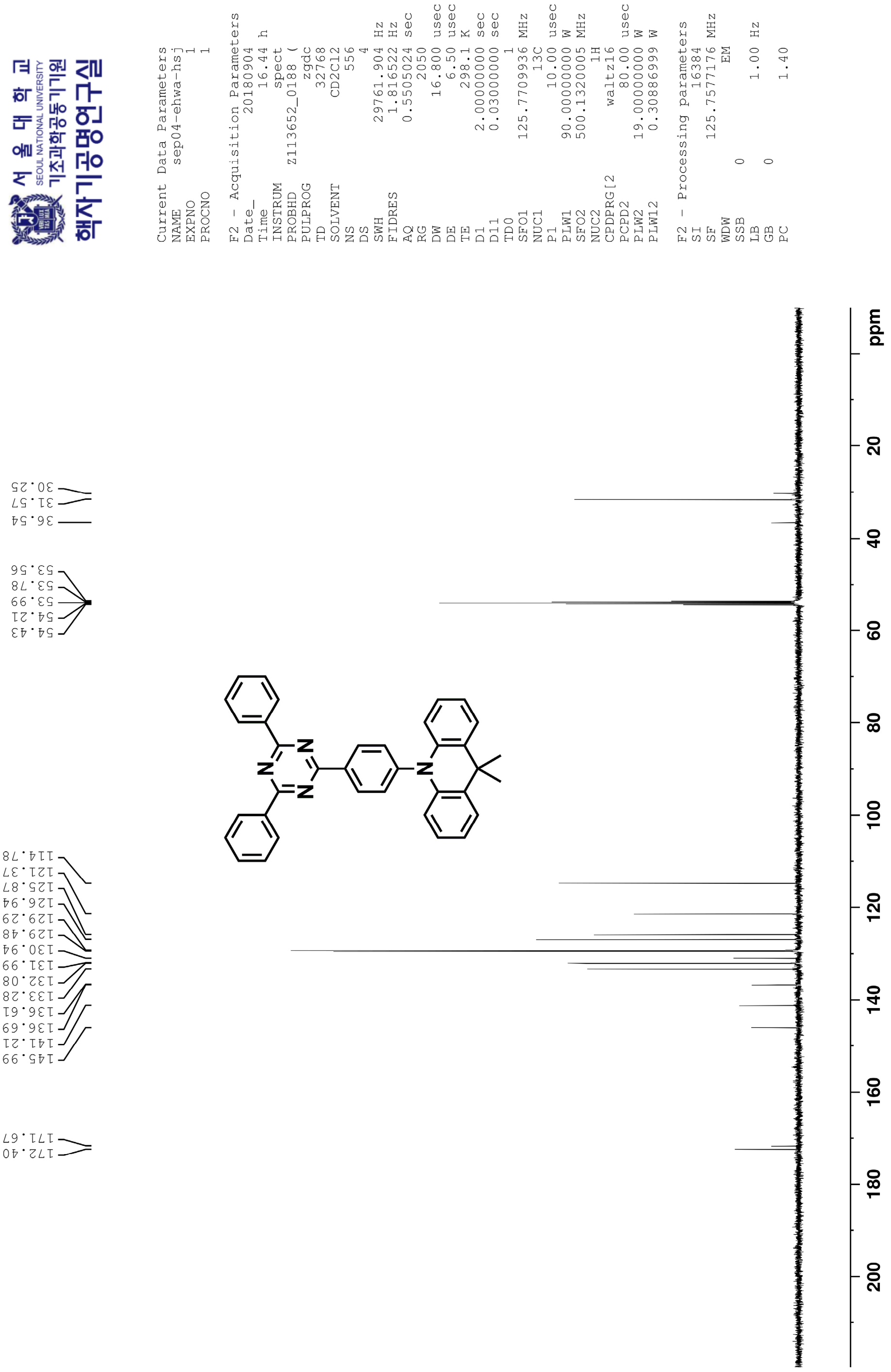


**Supplementary Figure 23.** $^{13}C\{^{1}H\}$ **NMR (126 MHz,** $CD_2Cl_2$**) spectrum of DMAC-TRZ.**

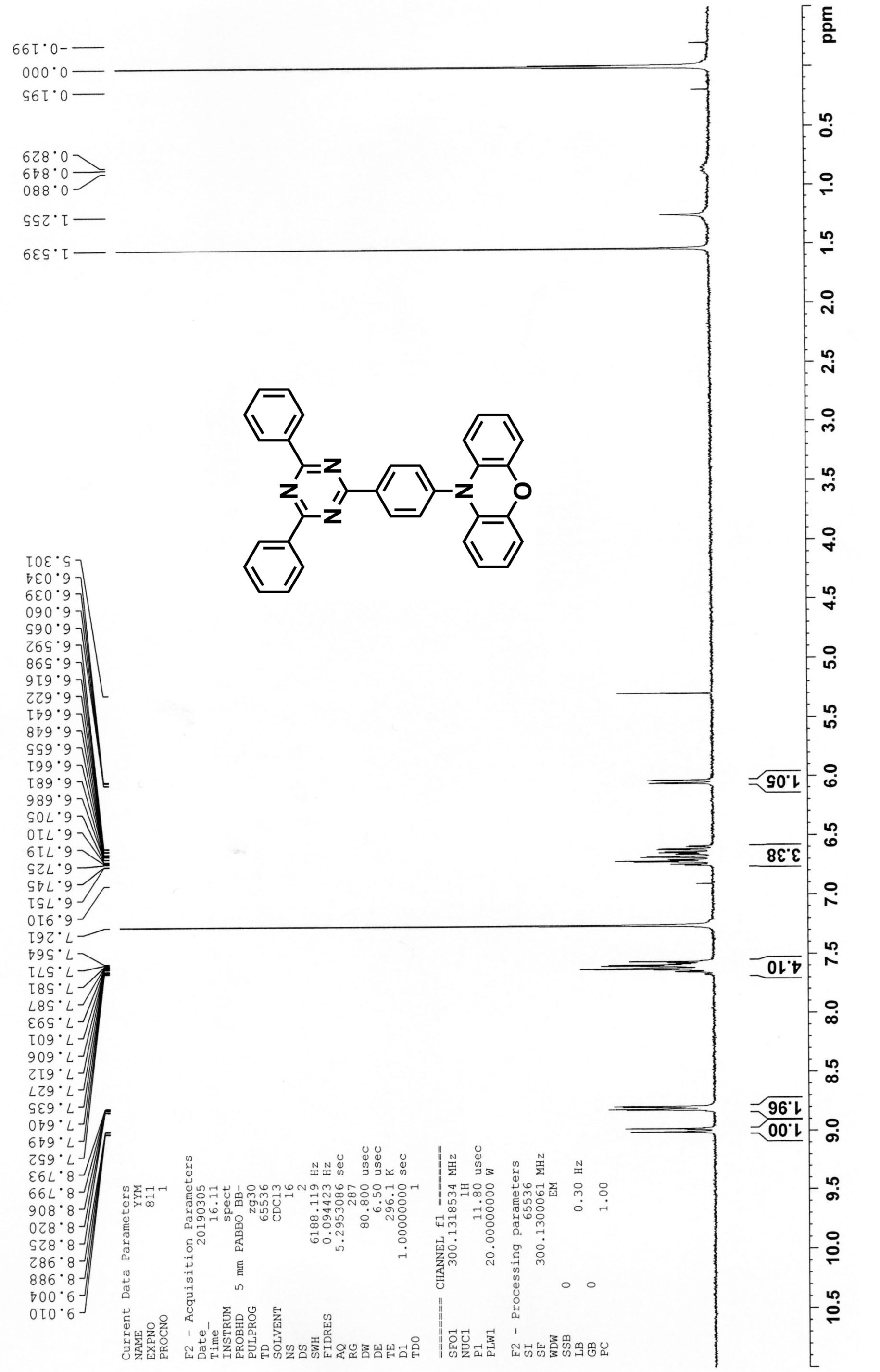

**Supplementary Figure 24. $^1$H NMR (300 MHz, $CDCl_3$) spectrum of PXZ-TRZ.**

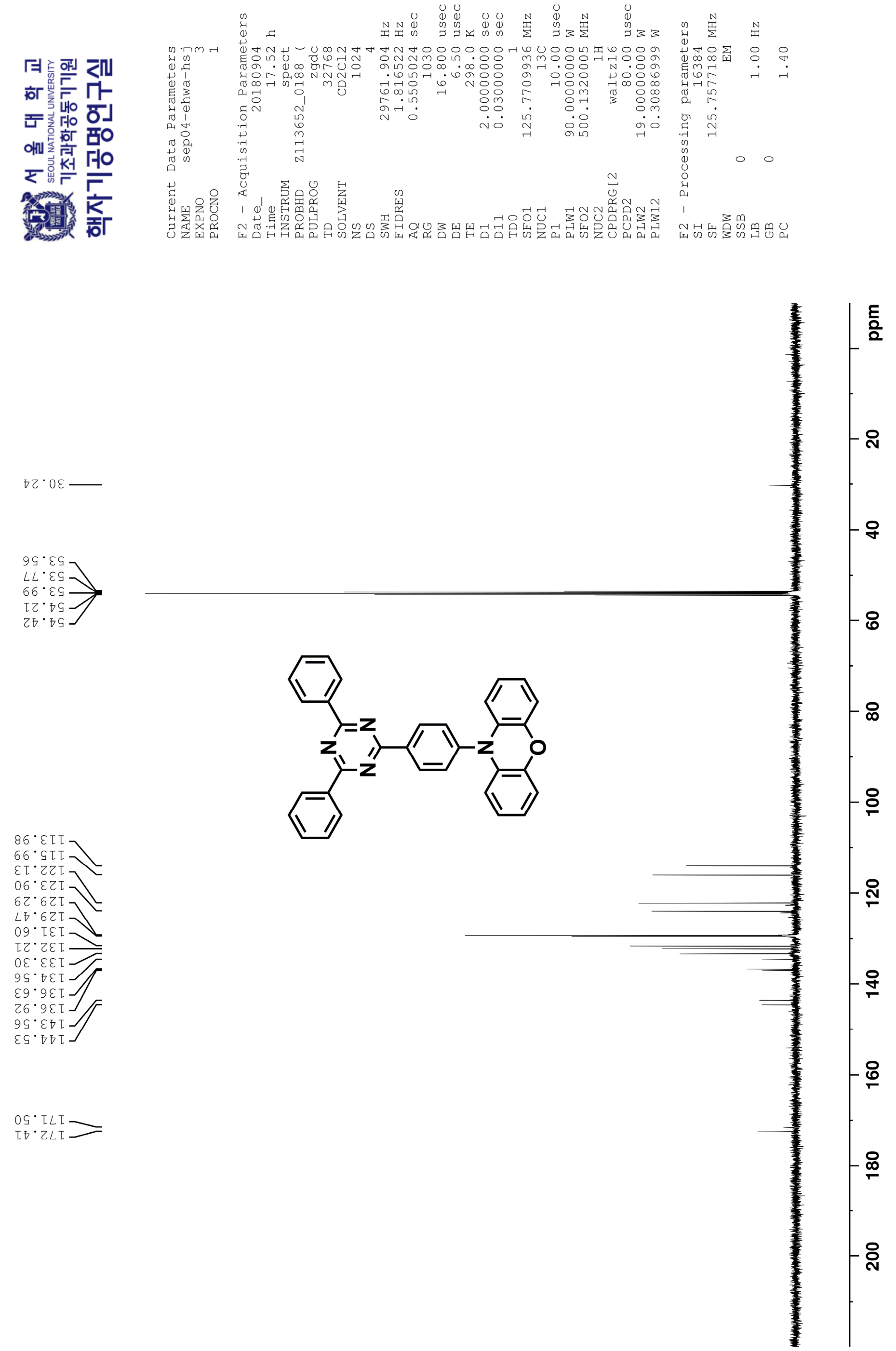


**Supplementary Figure 25.** $^{13}C\{^{1}H\}$ **NMR (126 MHz,** $CD_2Cl_2$**) spectrum of PXZ-TRZ.**

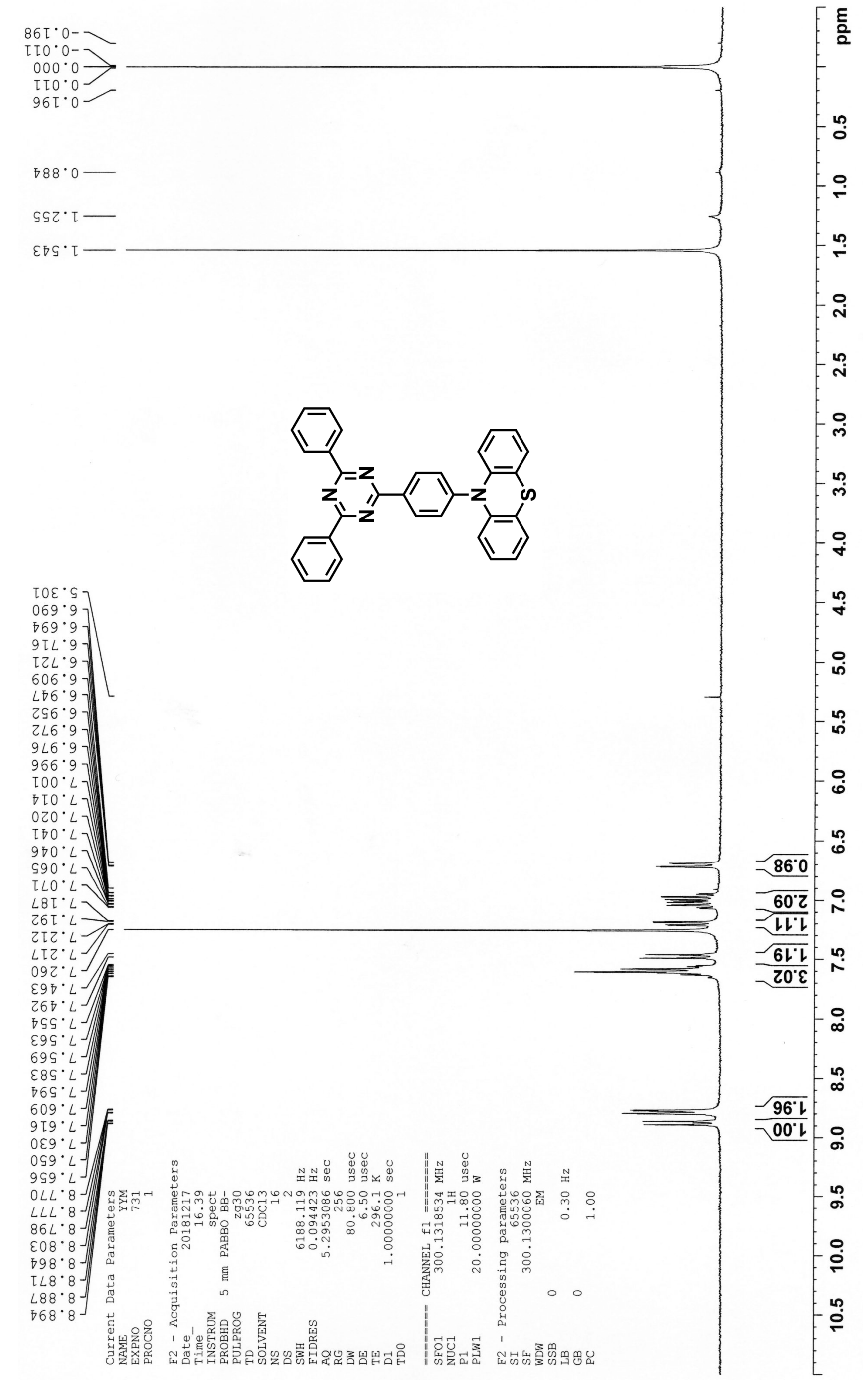

**Supplementary Figure 26.** **$^{1}$H NMR (300 MHz, $CDCl_3$) spectrum of PTZ-TRZ.**

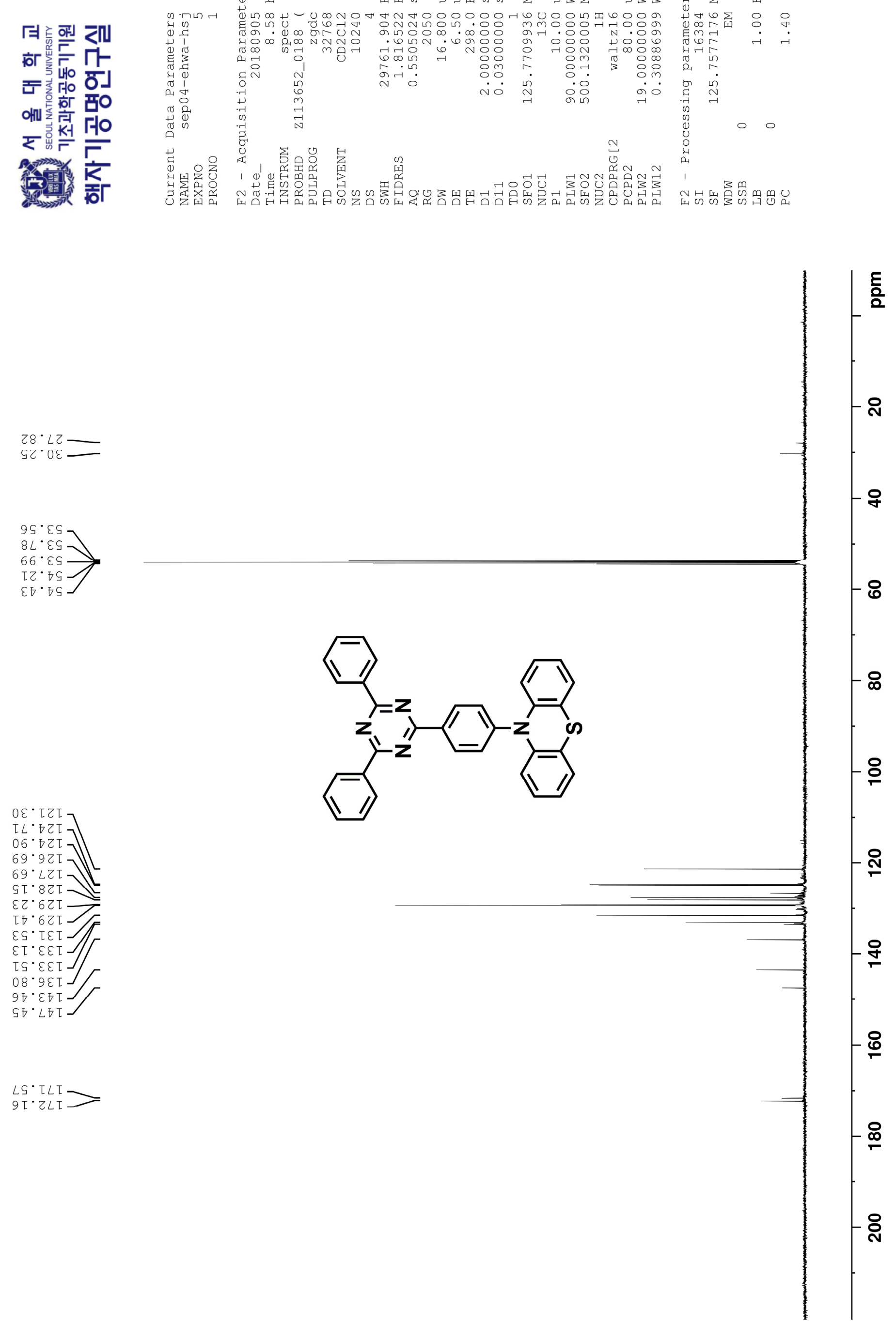

**Supplementary Figure 27. $^{13}C\{^1H\}$ NMR (126 MHz, $CD_2Cl_2$) spectrum of PTZ-TRZ.**

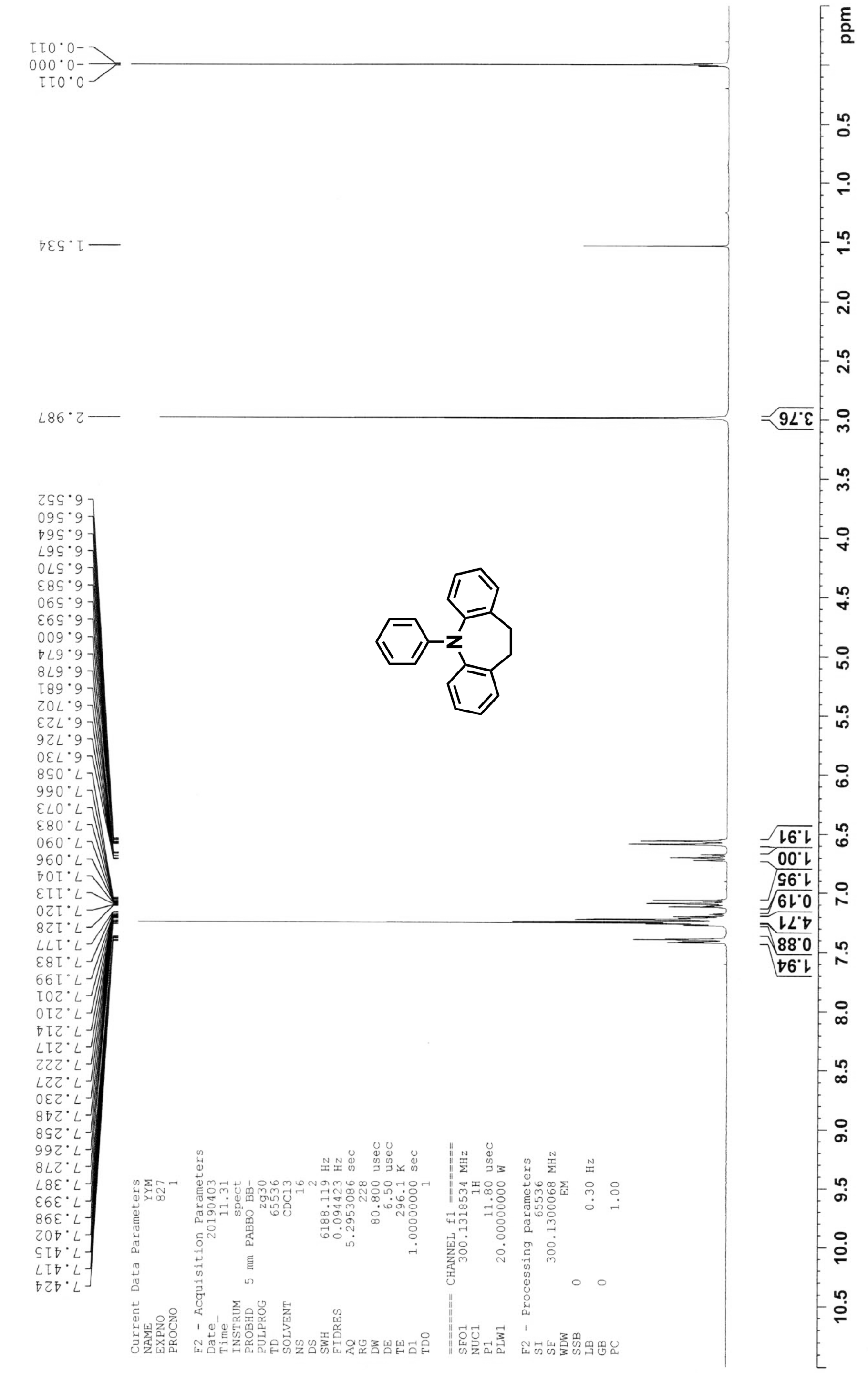

**Supplementary Figure 28. ¹H NMR (300 MHz, $CDCl_3$) spectrum of AZP-Ph.**

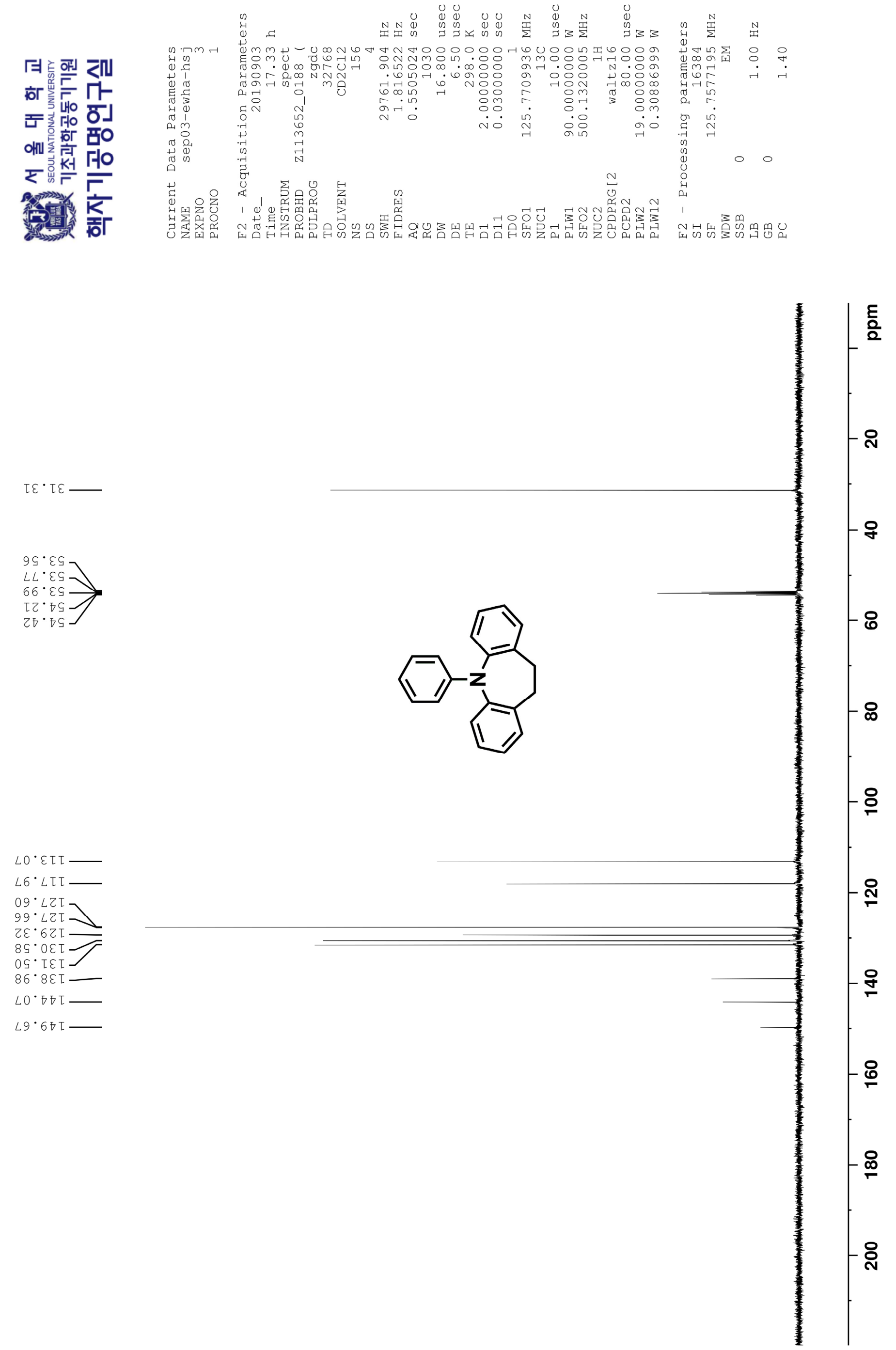


**Supplementary Figure 29.** **$^{13}C\{^1H\}$ NMR (126 MHz, $CD_2Cl_2$) spectrum of AZP-Ph.**

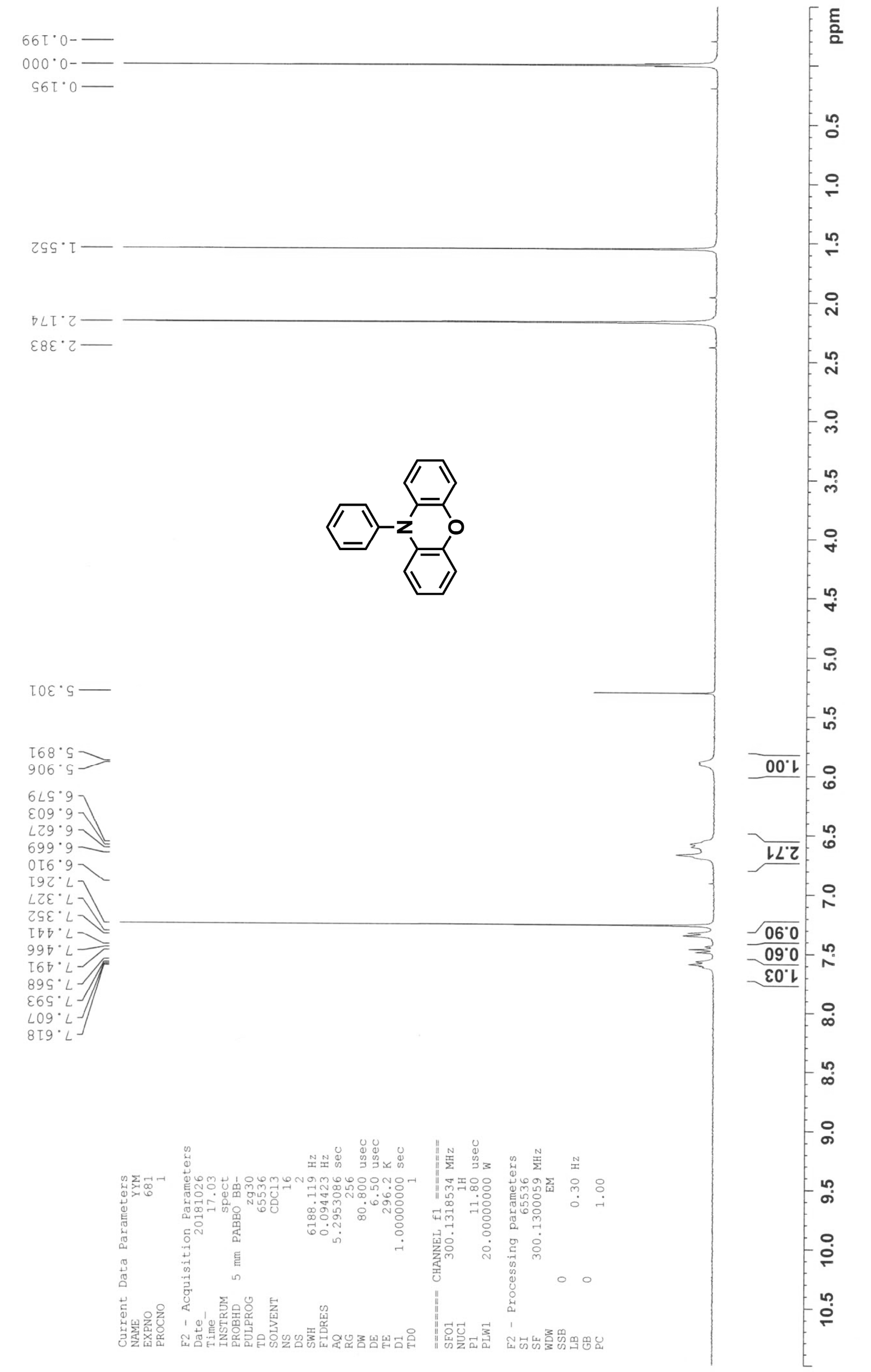

**Supplementary Figure 30. $^1$H NMR (300 MHz, $CDCl_3$) spectrum of PXZ-Ph.**

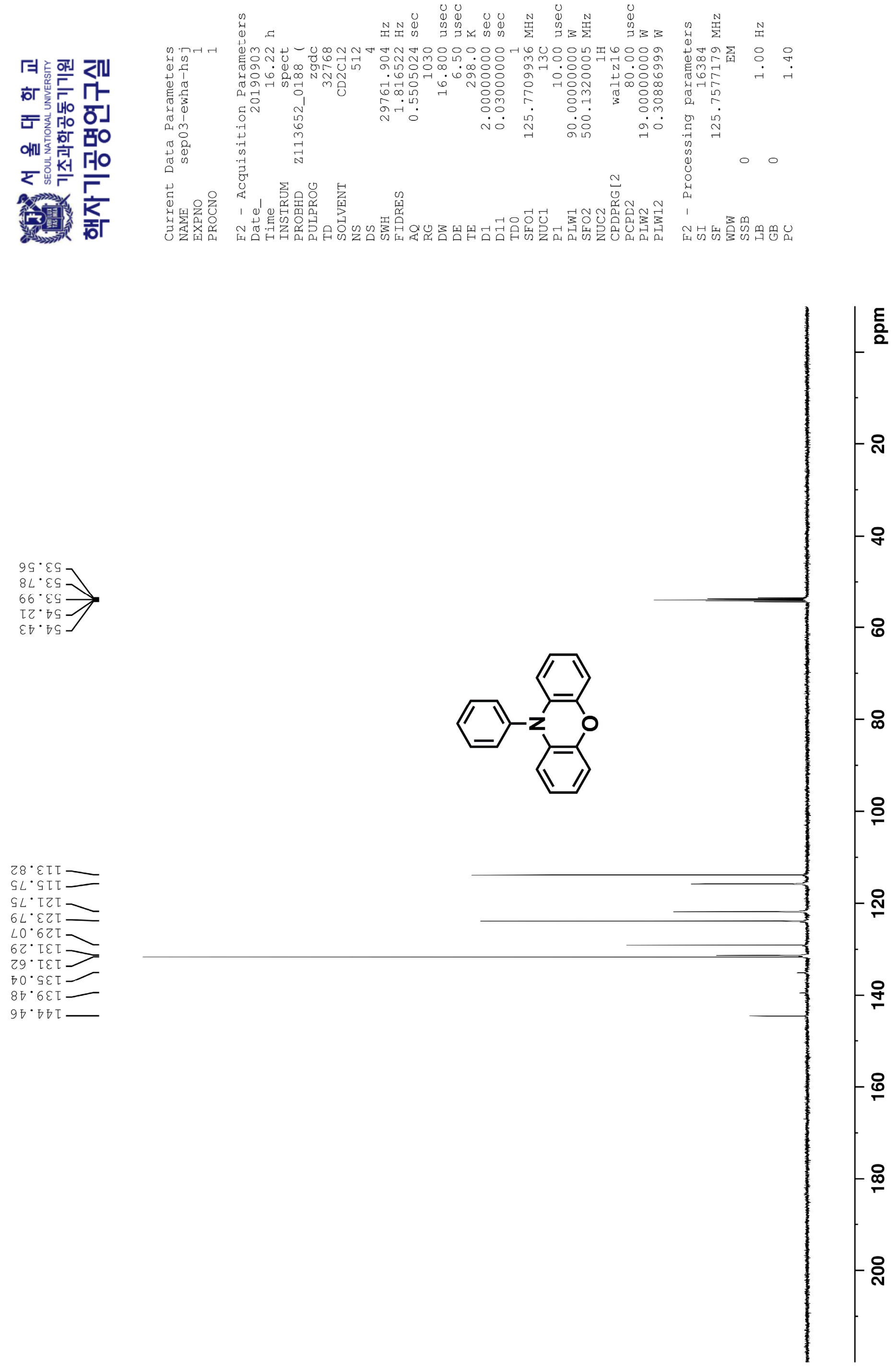


**Supplementary Figure 31.** $^{13}C\{^1H\}$ **NMR (126 MHz,** $CD_2Cl_2$**) spectrum of PXZ-Ph.**

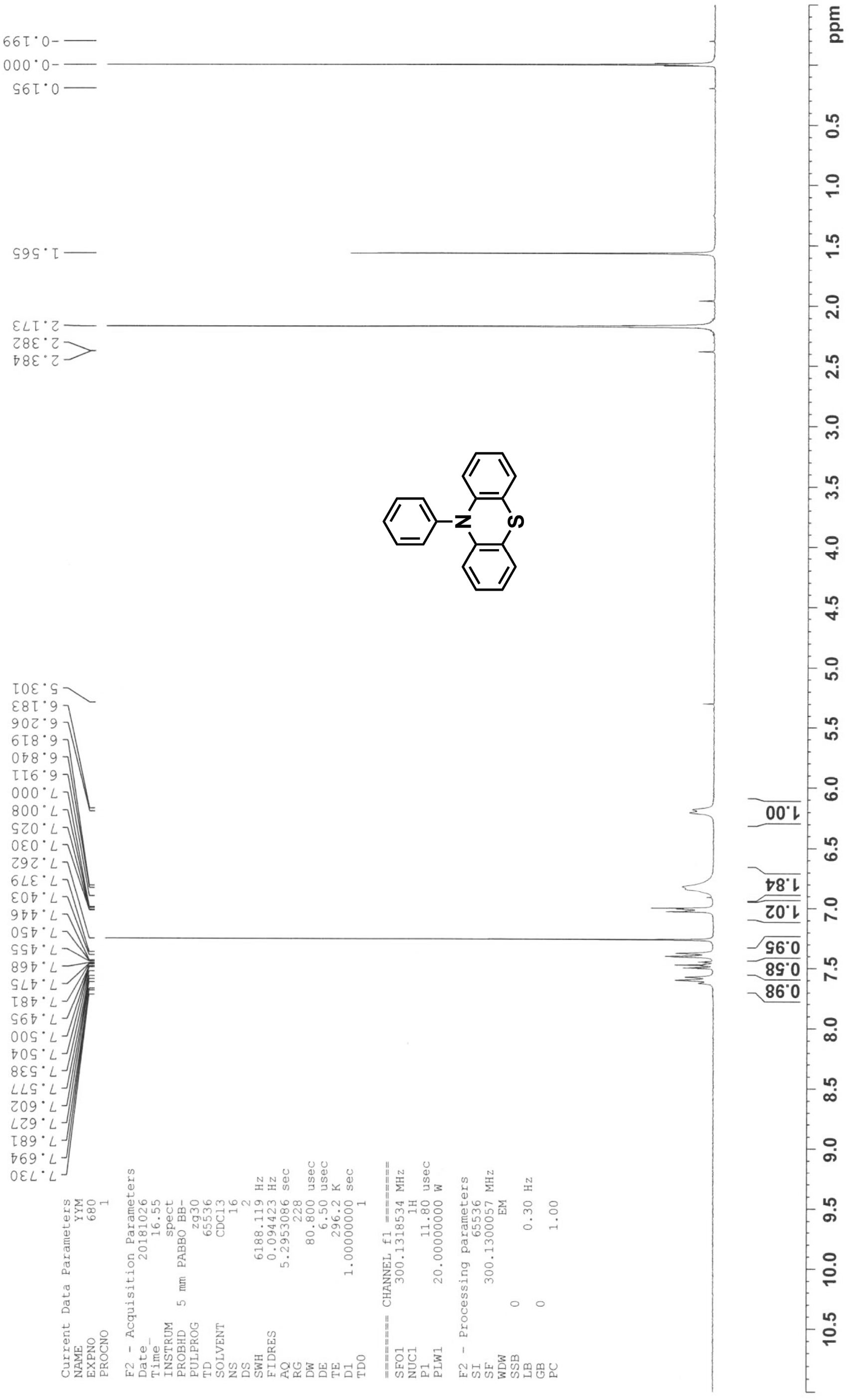

**Supplementary Figure 32. $^1$H NMR (300 MHz, $CDCl_3$) spectrum of PTZ-Ph.**

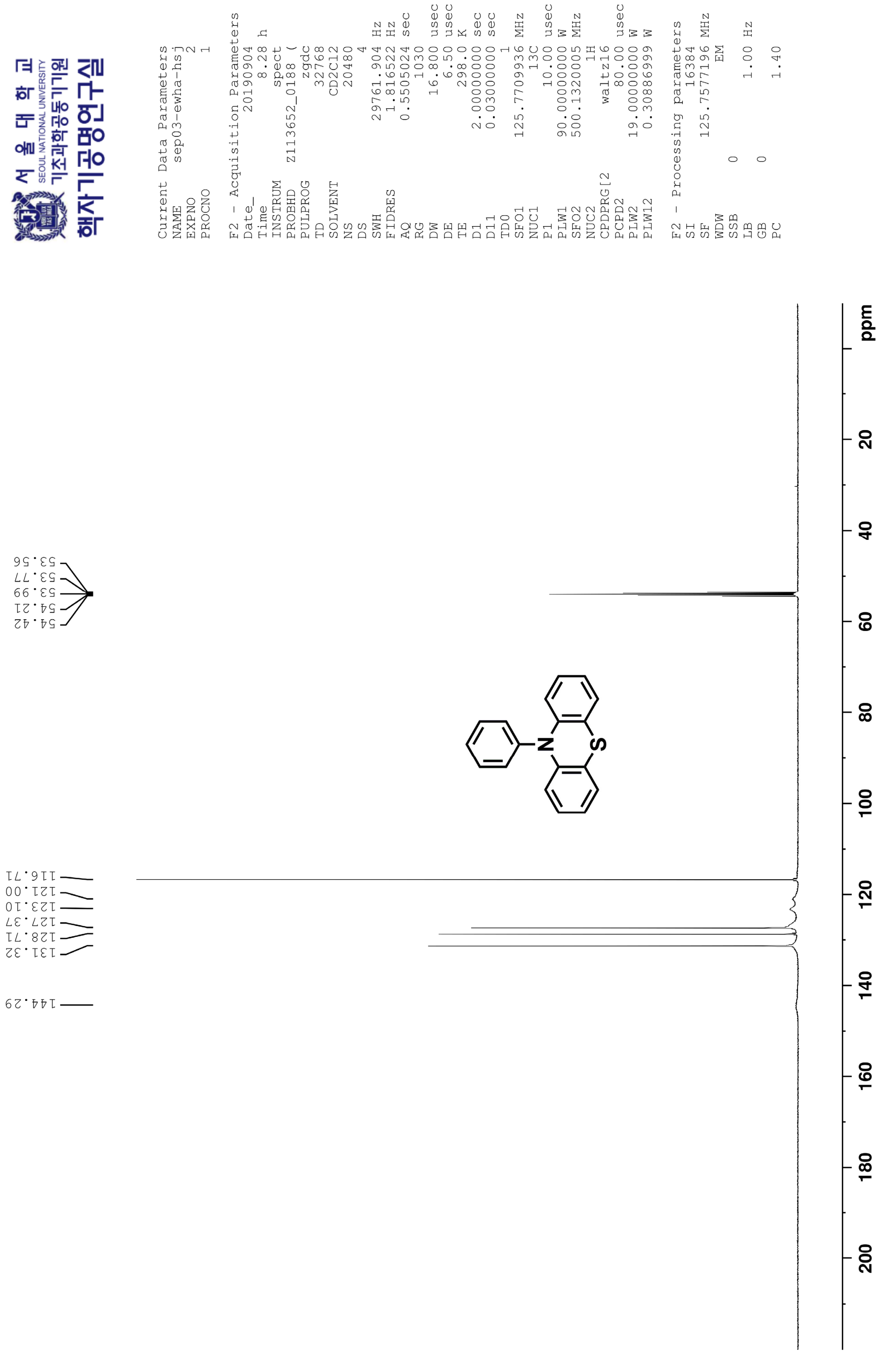


**Supplementary Figure 33.** $^{13}C\{^1H\}$ **NMR (126 MHz,** $CD_2Cl_2$**) spectrum of PTZ-Ph.**
