# Supplementary material for: Conformation-dependent degradation of thermally activated delayed fluorescence materials bearing cycloamino donors
Source: Commun Chem. 2020 May 1;3:53. doi: 10.1038/s42004-020-0303-4 (PMC9814945; doi:10.1038/s42004-020-0303-4)
Supplement: Supplementary file 3 — Description of Additional Supplementary Files [file 42004_2020_303_MOESM3_ESM.pdf]

Description of Additional Supplementary Files

File Name: Supplementary Data 1

Description: crystallographic information file for 10,11-dihydro-5*H*-dibenz[*d,f*]azepine 2,4-diphenyl-1,3,5-triazine (AZP-TRZ)
